# An automated modular open-technology device to measure and adjust concentration of aquatic sperm samples for cryopreservation

**DOI:** 10.1016/j.slast.2022.11.002

**Published:** 2022-11-29

**Authors:** Nikolas Zuchowicz, Yue Liu, W. Todd Monroe, Terrence R. Tiersch

**Affiliations:** a Aquatic Germplasm and Genetic Resources Center, School of Renewable Natural Resources, Louisiana State University Agricultural Center, Baton Rouge, LA, USA; b Department of Biological and Agricultural Engineering, Louisiana State University & Louisiana State University Agricultural Center, Baton Rouge, LA, USA

**Keywords:** Open technology, Concentration, Dilution, 3-D Printing, Aquatic, Cryopreservation

## Abstract

Repositories for aquatic germplasm are essential for safeguarding valuable genetic diversity for species relevant to aquaculture, biomedical research, and conservation. Development of aquatic germplasm repositories is impeded by a lack of standardization within laboratories and across the research community. Protocols for cryopreservation are often developed *ad hoc* and without close attention to variables, such as cell concentration, that strongly affect the success and reproducibility of cryopreservation. The wide dissemination and use of specialized tools and devices as open hardware can improve processing reliability and save costs. The goal of the present work was to develop and prototype a modular and open-technology approach to help to standardize the cell concentration of germplasm samples prior to cryopreservation. The specific objectives were to: 1) design and fabricate prototypes of the automated concentration measurement and adjustment system (CMAS), incorporating custom peristaltic pumps and optical evaluation modules, and 2) evaluate the performance of the CMAS with biological samples. Linear regression models were obtained for estimation of aquatic sperm concentration *>*10^8^ cells/mL and for algae concentration *>* (3 × 10^5^) cells/mL. Algae were diluted with extender medium by an automated process, resulting in a dilution precision of ±12.6% and ±6.7% in two trials, attaining means of 89% and 71% of the target cell concentration. The development of the CMAS as open technology can provide opportunities for community-level standardization in cryopreservation of aquatic germplasm and can invite new users, makers, and developers into the open-technology community. This will increase the reach and capabilities of much-needed aquatic germplasm repositories.

## 1. Introduction

Cryopreservation of animal germplasm has developed over the past half-century into a vital tool to support biomedical research, conservation, and agriculture [1,2]. In commercial-scale processing and human health care, strong financial and regulatory incentives have led to highly standardized processing of cryopreserved sperm samples [3,4]. The cattle industry is a well-developed model: frozen French straws of bull semen can be sold for thousands of US dollars each, and so collection, freezing, and storage methods are well established and carefully followed [5]. By contrast, in aquatic species it is common for each laboratory or germplasm bank to use its own locally developed methods [6–8]. Thus, a lack of standardization makes it difficult to reliably produce high-quality samples and to share them among sites; it also substantially decreases the utility and ease of use of large collections of germplasm that are drawn from many species and contributors [9].

This problem is particularly severe in species that are of little utility to agriculture and industry, but that are nonetheless ecologically important. In conservation, there is a pressing need to preserve the genetic diversity of imperiled species [10], but efforts are often hampered by modest funding, lack of trained personnel, and limited access to germplasm (e.g., owing to restricted or unpredictable breeding seasons [11]). The usual aim, to capture the greatest possible wild genetic diversity before it is lost, is different from that of the cattle model, where technicians can process adequate samples from a small number of desirable sires [5]. In addition, few aquatic protocols developed at the research level have been scaled up to commercial and industrial processes [12]. To achieve this scaling, protocols and hardware must be standardized. Development of a repository-support community could produce technologies to increase the throughput and reliability of cryopreservation and its related processes [13,14].

Conservation of coral genetic diversity gives a concrete example of how technology can assist processing for repositories. Corals generally have a restricted reproductive window amounting to a few hours each year over one, two, or a few days [15], and many species in the same geographical area may spawn on the same nights or even simultaneously [16,17]. It thus becomes a race to cryopreserve the sperm from dozens of distinct individuals of one or more species. In practice, each sample must be assessed for motility and concentration [18], diluted with cryoprotectant, distributed into containers, and frozen. These steps typically entail prolonged, manual handling. Samples are often wasted due to decline of quality before they can be processed. Automation of the handling steps could reduce waste, improve throughput, permit preservation from a greater number of individuals, improve the reliability, reproducibility, and audit trail of processes, and greatly increase the value of frozen samples.

Such challenges and opportunities cut across species and fields. Researchers working with fish and amphibian species have likewise identified the need for automation, especially in the pre-freeze processing steps, where the requirement may be for dozens of distinct samples to be evaluated and packaged into hundreds or thousands of containers in the space of a few hours. In one of a few high-throughput applications in aquatic species, the Aquatic Germplasm and Genetic Resources Center (AGGRC) has evaluated and cryopreserved the sperm of 50–100 channel catfish (*Ictalurus punctatus*) [19] or blue catfish (*Ictalurus furcatus*) males [20,21] over a three-day period. Executing this task requires a great deal of training, specialized equipment exceeding a total capital cost of US$250,000, and roughly 200 person-hours of effort. Customizable, inexpensive devices to improve efficiency, throughput, and reliability are urgently needed.

An explosion of open scientific hardware is delivering advanced, inexpensive, and customizable capabilities to a growing community of users, makers, and developers. Syringe [22] and peristaltic [23] pumps, photometers [24], and microfluidic and millifluidic elements [25,26] can be downloaded in digital form and 3-D printed in thermoplastic or photopolymerizable resin, laser cut, or assembled from commercially available parts. Specialized fields such as cryopreservation benefit from rapid dissemination of new, bespoke tools [27–30] that answer unusual but important problems, but that may not be suited to large-volume production by a major manufacturer.

The goal of the present work was to develop and prototype an automated, modular, low-cost, open-technology approach to measure and adjust the cell concentration of germplasm samples prior to cryopreservation, with the option to add necessary cryoprotective agents in the dilution process. The specific objectives were to: 1) design and fabricate prototypes of the automated concentration measurement and adjustment system (CMAS), incorporating custom peristaltic pump and optical evaluation modules, and 2) evaluate the performance of the CMAS with biological samples. The components of the CMAS were found to operate as a cohesive device, and the CMAS was found to be capable of estimating the concentration of catfish sperm and algal samples and diluting algal samples to a target cell concentration as a proof-of-concept for integration in a high-throughput cryopreservation workflow.

## 2. Methods

### 2.1. Fabrication of CMAS prototypes

**Fig. 1.**
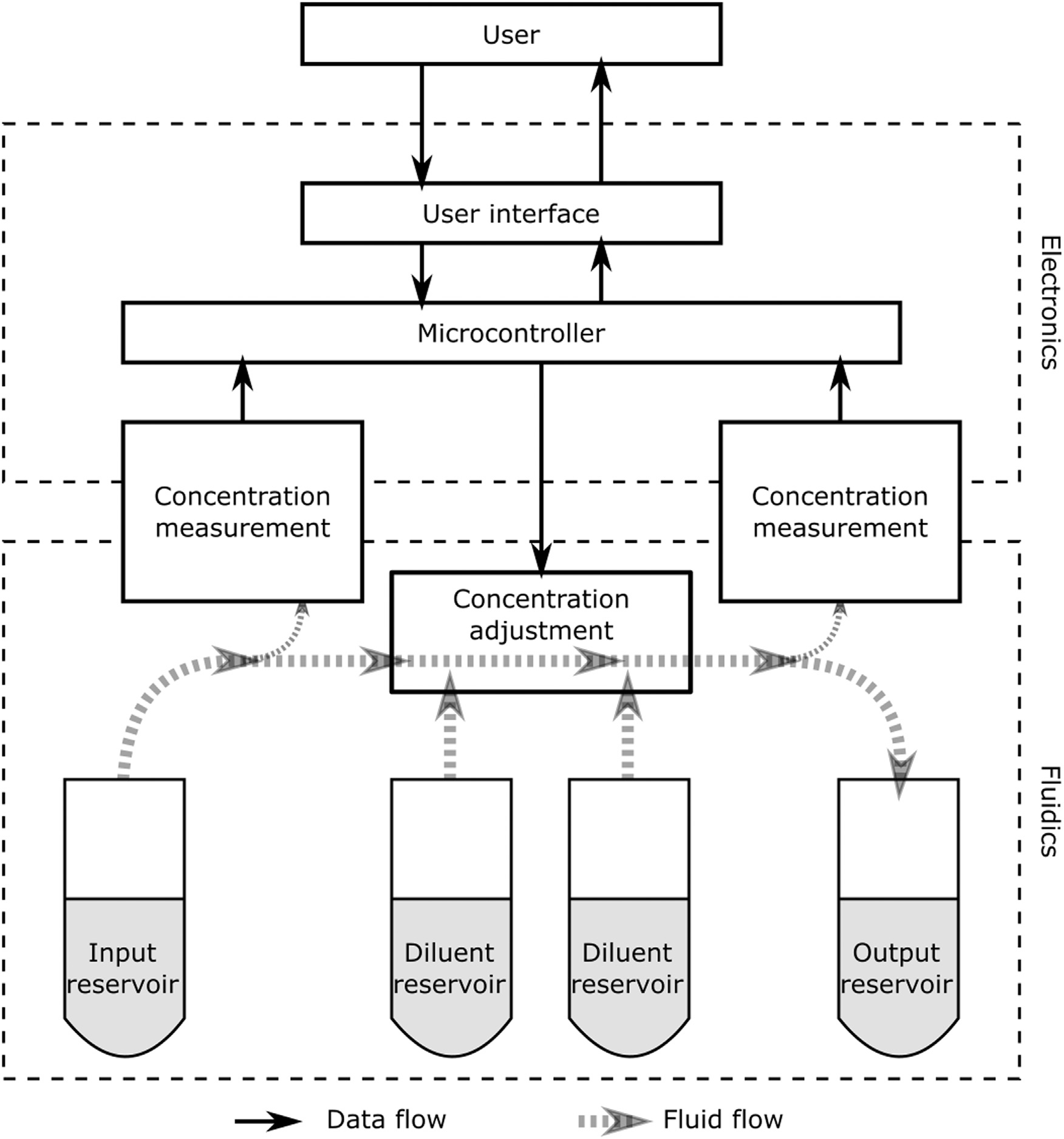
Design concept for the CMAS. A fluidic sample flows from the input to the output reservoir, with the measurement of sample concentration and the addition of diluents. The microcontroller mediates among the user, user-interface components, sensing components for concentration evaluation, and electromechanical components for fluid flow. The built-in concentration measurement after adjustment (at right) was not implemented in the present work.

The design concept of the CMAS was based on the concentration measurement and fluidic dilution functions normally performed manually in germplasm processing at the AGGRC [21] and elsewhere [7,8]. The flow of data and the movement and mixing of fluids are necessary to automate these functions (Fig. 1). An Arduino microcontroller platform mediated between the user, with a screen interface and buttons or rotary encoder, and the fluidic and sensing components of the device.

The fluidic flow consisted of an input reservoir containing the sample to be evaluated and diluted, one or more diluent reservoirs (e.g., 50-mL centrifuge tubes) containing extender medium or cryoprotectant medium, an output reservoir for the final, diluted sample, tubing to connect these, and pumps to move fluid through the tubing. Optical sensing modules at the beginning and end of the fluidic flow process were used to evaluate the concentration of samples before and after dilution. Evaluation at the beginning of flow was necessary to determine a mixing ratio to drive flow rates controlled by the pump to achieve a final target dilution. Evaluation at the end of the fluid flow was considered optional for the present proof-of-concept work; it may be added in future work as a built-in quality control measure to verify in real time whether the target concentration has been achieved.

The manual preparation of aquatic sperm for cryopreservation can comprise at least two dilution steps; the CMAS was designed with this serial dilution in mind. For example, the cell concentration might first be adjusted to 2 × 10^9^ cells/mL in an isotonic extender medium, then diluted 1:1 with a double strength (e.g., 20% v/v) cryoprotectant solution, for a final target cell concentration of 1 × 10^9^ cells/mL and final cryoprotectant concentration of 10% [21]. The fluidic system contemplated here has two diluent reservoirs that can be used to implement a process replicating these manual dilution steps.

Documentation of the assembly of the CMAS, the bill of materials, circuit diagrams, and Arduino code are provided in the supplementary materials.

#### 2.1.1. Device housing

The housing of the CMAS device was designed in Fusion 360 CAD software (version 2.0.10244, Autodesk, San Rafael, USA). The designs of the case frame were exported in .stl format to allow 3-D printing. The flat design patterns for the exterior panels of the CMAS case were exported from Fusion 360 to .dxf format, which contains vector data indicating the patterns to be fabricated by laser cutting.

All 3-D printed components were prepared in Cura slicing software (version 4.9.0, Ultimaker, Utrecht, Netherlands) and printed on an Ultimaker 3 Extended (Ultimaker) fused filament fabrication (FFF) printer. All components were printed in polylactic acid (PLA) thermoplastic (eSun, Shenzhen, China, esun3d.net). PolySupport filament (PolyMaker, Shanghai, China) was used as an underlying raft material to improve the bed adhesion and reliability of prints. Parts were printed at 0.15-mm layer height; full printer settings are provided in Suppl. Mat. F.

The .dxf files were imported into Inkscape, a free vector-image manipulation program, and exported to .pdf format, which can be used directly by the Muse 1064-nm CO_2_ laser cutter (Full Spectrum Laser, Las Vegas, Nevada, USA). Parts were cut from 6-mm-thick clear polymethylmethacrylate (PMMA, or acrylic) in vector cutting mode. Cutting was performed with three passes at 50% speed, 100% power, and 100% current. Multiple passes were employed to prevent overheating and scorching of the acrylic.

Sources for generic materials such as PMMA sheets, fasteners, standardized motors, and electronic components are provided in Suppl. Mat. B: CMAS Bill of Materials.

#### 2.1.2. Optics

**Fig. 2.**
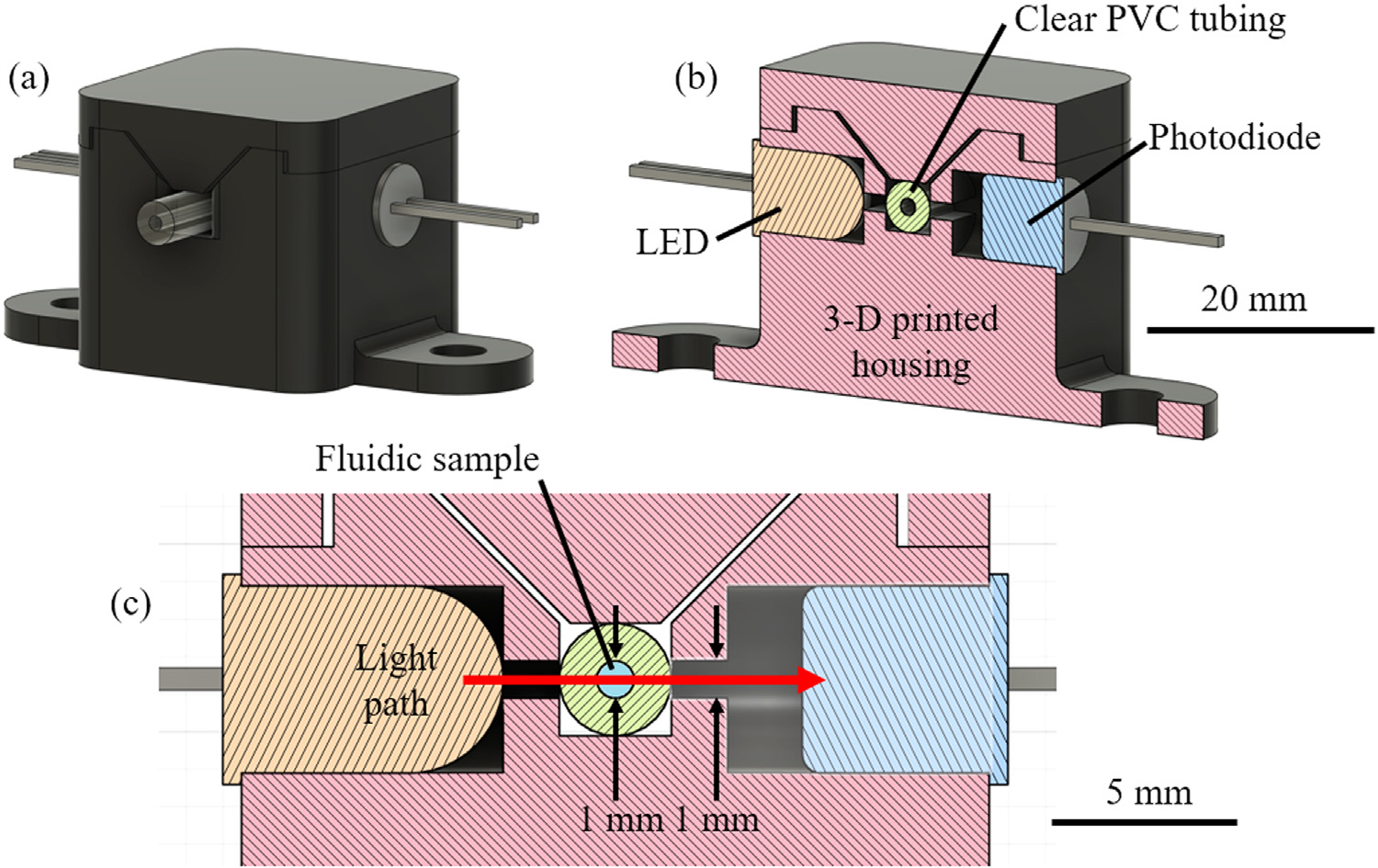
Illustrations of the optical enclosure. (a) The optics housing was designed to measure the intensity of light passing through a sample in clear PVC tubing. The LED, photodiode, and tubing were enclosed in a 3-D printed housing to exclude ambient light. (b) A cutaway illustration to show internal features. (c) Light (red arrow) passed from a light-emitting diode (LED), through the walls of the tubing and through the sample, to a photodiode. Note that the tubing internal diameter and the slit through which light was admitted were both 1 mm.

A custom optics housing was 3-D printed as an enclosure (Fig. 2). A three-color light-emitting diode (LED) (QT-Brightek 5-mm tri-color round lamp, part number QBL8RGB60D0–2897, qt-brightek.com) was chosen as the light source for the optical evaluation module. This LED had four electrical connections: a common anode and a separate cathode for each of the three colors. The LED was therefore able to produce red (according to the manufacturer datasheet, peak *λ* = 624 nm), green (*λ* = 525 nm), or blue (*λ* = 470 nm) light with changes only to connections in the electronics, rather than by changing the physical LED component. Only red light was used in this work, while the other two colors were left as options for future work. The emission spectra of the three LED colors were measured on a USB2000 UV-vis spectrometer (Ocean Insight, Orlando, FL, USA) by use of OceanView 2.0.8 software (Ocean Insight).

#### 2.1.3. Peristaltic pump

**Fig. 3.**
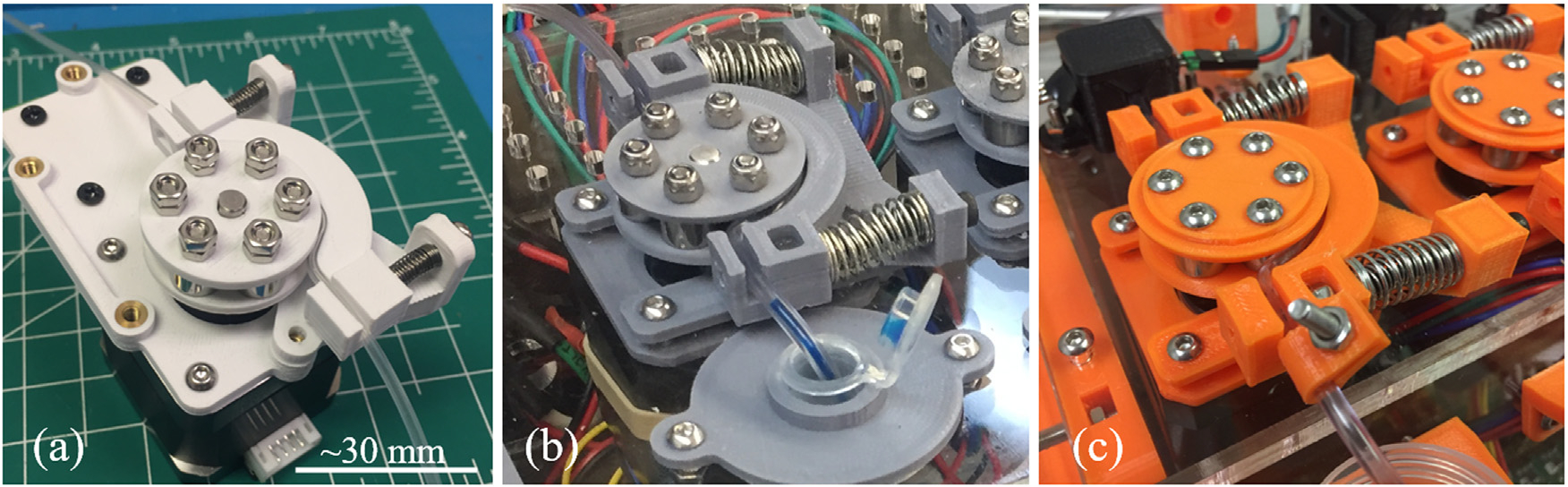
Three prototype iterations of the custom peristaltic pump for the CMAS. All versions were designed to be driven by a NEMA 17 stepper motor. (a) The first version was designed on a 3-D printed mount meant for attachment to metal DIN rails, common in networking and power cabinets. Scale bar is approximately accurate at the rotor (⌀ ~30 mm in all versions). (b) The second version featured stronger springs and was designed to mount on a laser-cut acrylic panel. (c) The third version featured a strengthened frame and a redesigned rotor assembly.

In the design of the peristaltic pump (Fig. 3), desirable features included freely rotating rollers, clamping features to hold the tubing in place, and flexibility in the type of motor used. The present design incorporated six roller bearings in the rotor. Compared to conventional three-roller pumps, this approach increased the number of points of contact between the rotor and the tubing. This in turn allowed the tubing to pass directly from one side of the pump to the other, eliminating the requirement to have the tubing wrap around the rotor completely, entering and exiting the pump on the same side, as is the case in some existing designs [23,31]. The pump was designed to fit a NEMA 17 stepper motor (StepperOnline, Nanjing City, Jiangning, China), chosen for its high torque and precisely controllable rotation speed. The National Electrical Manufacturers Association (NEMA) size 17 (1.7” × 1.7” faceplate) standard for stepper motors encompasses a set of motors of varying design, torque, and manufacturer, so motors of different specifications may easily be substituted.

Components of the pump were 3-D printed with the addition of PolySupport filament as support material under the pump rider arms to address overhanging geometries. For printing on a single-nozzle 3-D printer, this support structure could equally be printed from the same material as the pump rider arms, i.e., PLA thermoplastic.

No dedicated millifluidic or microfluidic mixing device was integrated into the tubing. Instead, the fluid flows were brought together in simple T-junctions. This presented no practical problem in mixing, since the output vial should be swirled briefly by the user in any event before drawing off sample for cryopreservation to ensure even suspension of the cells in solution. This simple mixing action matched that normally performed in manual dilution.

#### 2.1.4. Electronics

Electronics of the CMAS were assembled on soldered breadboard; the full circuit diagrams are provided in Suppl. Mat. C. An A4988 stepper motor driver board (BIQU Equipment, Shenzhen, Guangdong, China) was used to actuate each NEMA 17 stepper motor. This board acted as a convenient buffer between the 5-V Arduino logic and the 12-V motor power supply. The driver board also handled the four-wire control sequence required by the stepper motors, presenting to the microcontroller a simple two-wire interface consisting of a ‘step’ line, which was pulsed to increment the position of the motor shaft, and a ‘direction’ line, which directed clockwise or counter-clockwise rotation.

To run three stepper motors along with the electronics, more power was required than could be provided by many common consumer-level AC–DC power adapters. Power was therefore supplied by a generic 360-W (12-V, 30-A) switching power supply typically used to power 3-D printers and similar projects. One 12-V output from the power supply was connected directly to the A4988 stepper motor driver boards to power the stepper motors. A second 12-V output was routed to an LM7805 linear voltage regulator to supply 5 V to all other electronic components.

Because Arduinos have built-in analog-to-digital converters (ADC) but no digital-to-analog converters (DAC), a MCP4725 DAC IC (Microchip Technology, part MCP4725AX) was used to drive each LED, converting a 12-bit digital input signal from the Arduino into an analog voltage to run the LED. Each MCP4725 achieved a voltage resolution of about 1.2 mV (5 V / 4095, where 5 V is the supply voltage) and the input signal could take any of 2^12^ = 4096 discrete states [32].

To quantify the light signal, a SFH 203 photodiode (Osram Opto Semiconductors, osram-os.com) was included in a reverse bias configuration [25]. An MCP6002 operational amplifier (Microchip Technology) was used with a gain factor of 20 × before ADC on the Arduino.

A DS3231 real-time clock breakout board (Adafruit Industries, adafru.it) was added to allow the logging of time-stamped events to the microSD card. This clock IC offered several advantages: a simple I^2^ C communication interface to the microcontroller; an onboard CR1220 coin cell battery (3 V) that can run the clock for several years without external power; correction for environmental temperature; and minimal time drift (min per year). The present firmware does not implement logging to the microSD card, but it is provided in the design for ease of integration as a future capability.

Custom firmware for the CMAS was written to mediate among the various electronic and electromechanical components. The firmware was written in the Arduino variant of the C++ programming language within the Arduino integrated development environment, version 1.8.13 (arduino.cc/en/software). The architecture of the firmware followed finite-state machine principles (Suppl. Fig. E.1) to promote modularity and to help prevent locking the software into infinite loops during runtime. The full Arduino code is provided in Suppl. Mat. D.

User interface components were included to allow the user to read data from the device and control the pumping and measurement functions. A 20 × 4-character LCD screen, a push-button/rotary encoder with a Schmitt trigger debounce circuit to remove mechanical button noise, and a microSD card breakout board were added as user interface components.

### 2.2. Evaluation of the performance of the CMAS with biological samples

#### 2.2.1. Catfish sperm samples: concentration estimation

To evaluate the sensitivity and performance of the CMAS, sperm samples were obtained from channel catfish, *Ictalurus punctatus*. Hanks’ balanced salt solution (HBSS) was prepared [33], and its osmolality was measured and adjusted to 301 mOsm/kg by use of an Osmette III freezing point osmometer (Precision Systems, Natick, Massachusetts, USA). Male fish were supplied on June 2 and 3, 2021 by the Warmwater Aquaculture Research Unit, United States Department of Agriculture, Stoneville, Mississippi, transported to Baton Rouge, Louisiana, and processed on the same day. All animals were handled in accordance with protocols approved by the LSU AgCenter Institutional Animal Care and Use Committee. The catfish were killed by a blow to the neurocranium. Testes were removed, cleaned (removing excess tissue and blood clots), weighed, and placed in a small plastic bag with HBSS (1 mL/1 g of testis mass). The testes were folded into a small square of window screen wire mesh and crushed inside the plastic bag until coherent tissue was no longer visible, thereby releasing sperm into the HBSS. The mesh was removed with the bulk of the crushed tissue inside it.

Sperm suspensions were filtered through a 100-μm filter into 50-mL Falcon tubes. Samples from each male were kept in separate tubes, each marked with a tracking number. A small aliquot for evaluation was removed and diluted in a ratio of 100:1 (HBSS:sperm solution). The diluted aliquot was pipetted into a Makler^®^ counting chamber (SefiMedical Instruments, Haifa, Israel; sefimedical.com) and the concentration of sperm of the undiluted sample was estimated by visual counting of the diluted samples and calculation. For the channel catfish assessed (n = 54), the body mass was 2.35 (SD, 0.79) kg, the body length was 50 (SD, 5) cm, the initial sperm concentration was 3.1 (SD, 1.4) × 10^9^ cells/mL, and the mass of dissected testes was 8.95 (SD, 4.34) g.

To evaluate the response of the optical evaluation module to different sperm concentrations, a series of dilutions of sperm samples was prepared from a single catfish (fish tracking number CCFH21M027, ‘Belle Prairie’ origin, body mass 1.77 kg, body length 46 cm, mass of dissected testes 9.62 g). This fish was chosen because it provided a high concentration of sperm, a large volume of sample (*>*30 mL) in excess of that required for cryopreservation and other experiments, and low contamination with blood, as determined by visual inspection of the sample color, compared to other samples. Calculating from the diluted aliquot, the undiluted concentration of this sample was estimated to be 4.0 × 10^9^ cells/mL as described above. Ten 0.5-mL samples were prepared, each of a different cell concentration. The first was an aliquot of the undiluted sample; the second sample and onward were prepared by 1:1 serial dilution of undiluted sample with HBSS. The ten serially diluted samples were therefore estimated to have nominal concentrations of 4.0 × 10^9^, 2.0 × 10^9^, 1.0 × 10^9^, 5.0 × 10^8^, 2.5 × 10^8^, 1.3 × 10^8^, 6.3 × 10^7^, 3.1 × 10^7^, 1.6 × 10^7^, and 7.8 × 10^6^ cells/mL.

Clean, empty polyvinyl chloride (PVC) tubing (McMaster-Carr item 3774N1, durometer 55A, inner diameter 1 mm, outer diameter 3 mm) was inserted into the optical evaluation module. The brightness of the LED was calibrated by manually adjusting the voltage produced by the DAC. The aim was to produce a baseline output voltage from the operational amplifier (i.e., the output voltage with empty tubing) of 1.50 V. This value was chosen from testing (data not shown) indicating that using this voltage as a starting point, a sample of deionized water produced an output voltage of approximately 4.1 V, while a sample of undiluted blue food dye (Spice Supreme food color, Gel Spice Co., Bayonne, New Jersey, USA), which permits little transmission of red light, produced an output voltage of approximately 0.5 V.

It was anticipated that all practical fluidic samples would fall between the optical density of deionized water and that of undiluted blue food dye. With these settings, no practical sample would saturate the bottom or top of the output range (i.e., approach 5 V or 0 V). Such saturation would make it impossible to distinguish between samples of different concentration within the saturated range. The ten diluted sperm samples were pumped through the optical module (one ‘pull’ from each sample tube from highest to lowest concentration, and this series was repeated four times) and the resulting signals were recorded. Between samples, HBSS was used to flush the tubing. The recorded signals were plotted against the cell concentrations calculated from the dilution ratios, and a best-fit linear regression equation was determined in Excel software (Microsoft Excel for Microsoft 365 MSO 16.0 64-bit).

To verify the predictive functionality of the equation obtained by linear regression, a new set of ten dilutions of sperm from the same male catfish was prepared, with two replicates of each. These samples were labelled and randomized by a collaborator. The correspondence between sample number and ‘known’ concentration was recorded but hidden from the operator (i.e., a single-blinded evaluation). The samples were evaluated with the CMAS and the concentration of each sample was estimated by use of the equation determined above. The CMAS estimates were plotted against the ‘known’ concentrations.

#### 2.2.2. Unicellular algal samples: concentration estimation and automatic dilution

Samples of the unicellular algae *Tetraselmis chuii* were used to evaluate the overall functioning of the final version of the CMAS. These microalgae are important as larval feed for aquatic organisms and like numerous other algal species are deserving of cryopreserved storage in repositories. The algal culture was obtained from Algae Research and Supply (Carlsbad, California, USA; algaeresearchsupply.com). The cell concentration of a sample of the culture was evaluated in the Makler^®^ chamber with an Axio Lab.A1 phase microscope (Zeiss, Oberkochen, Germany) and calculated to be 3.6 × 10^6^ cells/mL.

The optical absorbance of the algal sample across the visible spectrum was measured on a Nanodrop 1000 spectrometer (Thermo Fisher Scientific) in UV-vis mode. The absorbance was plotted with the LED emission distributions from the optical module.

Serial 1:1 dilutions of the algal culture were prepared with artificial seawater (32 ppt, Reef Crystals Reef Salt, Instant Ocean, instantocean.com) for a total of eight samples of calculated concentrations 3.6 × 10^6^, 1.8 × 10^6^, 9.0 × 10^5^, 4.5 × 10^5^, 2.3 × 10^5^, 1.1 × 10^5^, 5.6 × 10^4^, and 2.8 × 10^4^ cells/mL. Optical signals from the eight samples were derived in the same way as described for the channel catfish sperm samples in Section 2.2.1. The eight diluted samples were pumped through the CMAS (one ‘pull’ from each sample tube from highest to lowest concentration, and this series was repeated four times) and the resulting signals were recorded. Between samples, artificial seawater was used to flush the tubing. A linear regression was fitted to allow the calculation of the corresponding inverse function, and so to allow the determination of an unknown cell concentration from its optical signal.

To allow comparison of the optical performance of the CMAS to that of an existing commercial instrument, the absorbance of the same eight serially diluted samples was measured on a Nanodrop 1000 spectrophotometer at *λ* = 625 nm, corresponding to the *λ* = 624 nm peak of the red channel of the LED provided on the CMAS. The Nanodrop absorbance data were transformed to transmittance data by the formula

$$\%\;transmittance=10^{\left(2-absorbance\right)}\text{.}$$

To allow the automatic adjustment of cell concentration, the CMAS firmware was configured to calculate the concentration of samples pumped through the optical module. The function required to calculate the concentration was determined on a personal computer in Excel software. The CMAS was configured to automatically dilute an algal sample to a concentration of 1 × 10^6^ cells/mL. The CMAS calculated the appropriate dilution factor, the ratio between flow from a reservoir of artificial seawater and flow from the algal sample, to reach the final desired concentration. The function of this basic workflow was evaluated by drawing an aliquot of the original sample (3.6 × 10^6^ cells/mL) into the CMAS; this was repeated for a total of five trials. A 1:1 dilution of the original sample was prepared by pipetting, for a starting concentration of 1.8 × 10^6^ cells/mL; this was also repeated for a total of five trials. The resulting ten diluted samples were evaluated by eye with the Makler^®^ chamber to determine the final concentrations, and these concentrations were plotted.

## 3. Results

### 3.1. Fabrication of CMAS prototypes

**Fig. 4.**
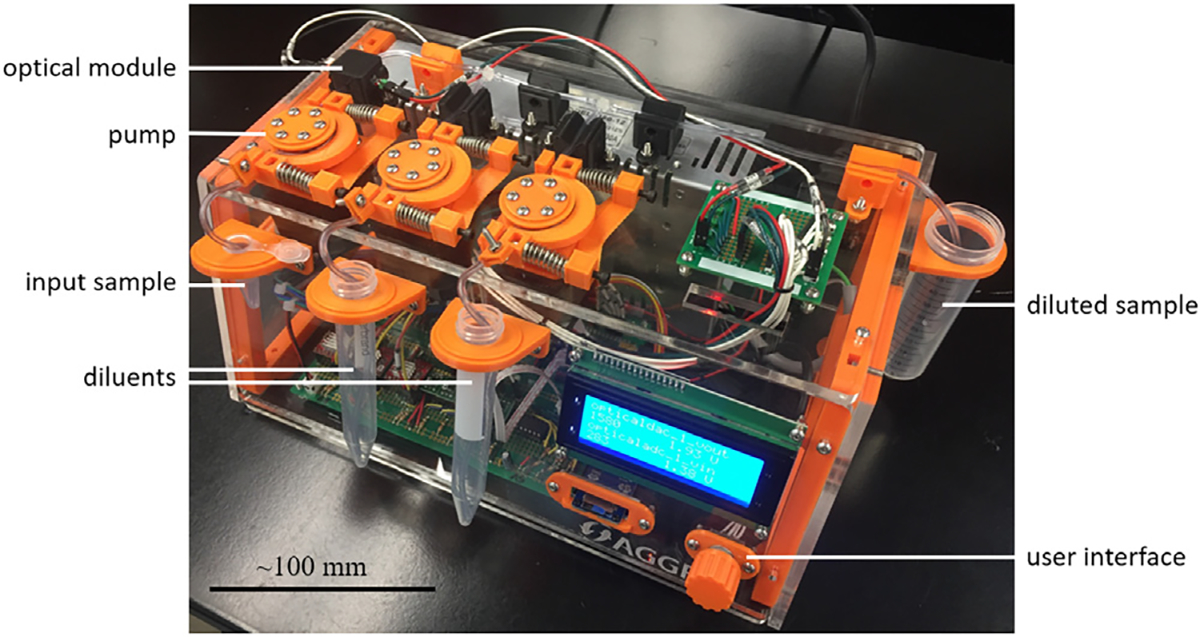
The third CMAS prototype. The input sample is pumped through an optical module, which estimates the sample sperm cell concentration. The diluents are then pumped into tubing T-junctions that meet the sample flow in a ratio calculated to achieve a target dilution, and the mixed fluid stream is produced into the diluted sample container. Scale bar is approximately accurate at the center of the front face of the case.

Design decisions were influenced by the results of an iterative prototyping process [27], in which three complete versions, with multiple modifications to individual components, were fabricated and evaluated (Fig. 4 and Suppl. Fig. E.4). The design of the peristaltic pumps was refined, with a more robust base to prevent material fatigue and failure, and with small modifications to the shape of the rotors to have them ride more smoothly on the tubing. The final pump design was found to pump up to 1.1 mL/min with the current electronics. Pumping rates did not vary with test solutions varying in viscosity from water (~0.96 cP) to 20% glycerol in water (~1.68 cP) [34], a plausible upper viscosity bound for a conventional cryoprotectant solution intended for bulk cryopreservation of germplasm by equilibrium freezing. Systematically labeled wire bundles (Suppl. Fig. A.4) were prepared to run between the various electronic components inside and outside the case, which simplified troubleshooting and reconfiguration. The brackets for the sample tubes were moved from the top of the enclosure to the outside front of the enclosure to prevent spilled sample fluid from entering the case.

The finished prototype (Fig. 4) comprised a solid enclosure of acrylic panels built onto a 3-D printed frame. This approach combined the flexible design options allowed by 3-D printing with the rapid fabrication of large design elements allowed by laser cutting. A completely 3-D printed enclosure of equivalent size and durability would have taken more than 24 h to print on most consumer-grade printers, whereas this enclosure was fabricated in under 6 h, including printing and laser-cutting time. Lessons from earlier prototype versions improved the safety and function of the device: solderless designs were committed to soldered breadboard, sample tube holders were moved to the exterior of the case to mitigate the danger of spills, and an AC–DC power supply was incorporated into the case.

The costs of materials to build the CMAS are detailed in Suppl. Mat. B. The cost of construction of a single CMAS as of 2021 was estimated to be US$560, not including taxes, shipping, tools, or fabrication equipment. If purchase of all tools and fabrication equipment was included, the cost was $4569. The bulk of this amount was to buy a laser cutter ($3524), which is not strictly needed. With the use of alternative fabrication techniques that may already be available (e.g., drill press and table saw) to fabricate the panels that constitute the case of the CMAS, the one-time cost of tools and equipment can be reduced to $483.

### 3.2. Evaluation of the performance of the CMAS with biological samples

#### 3.2.1. Catfish sperm samples: concentration estimation

**Fig. 5.**
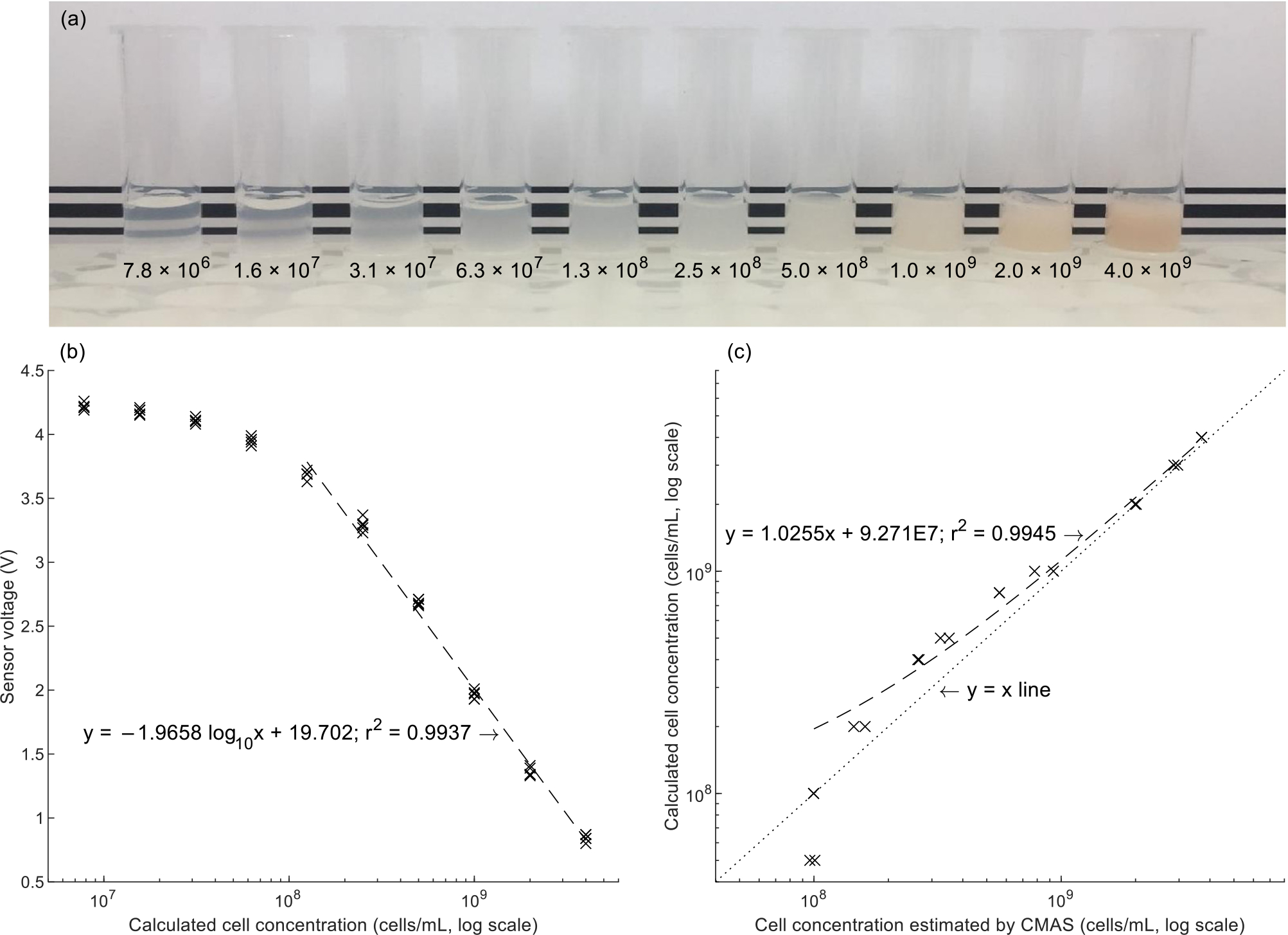
Optical measurements on the CMAS of serial dilutions of channel catfish sperm allowed fitting of a regression line. (a) Serial dilutions of sperm are shown, ranging from most dilute (7.8 × 10^6^ cells/mL) at left to the starting concentration (4.0 × 10^9^ cells/mL) at right. A faint red discoloration is visible in the high-concentration aliquots due to blood present in the sample, a normal artifact of the collection method. (Blood content ≤5% v/v changed the concentration estimates by *<*2%.) A sheet of paper with black lines was placed in the background to help to demonstrate the increasing opacity of the samples. (b) Sensor voltage from the CMAS optical evaluation module was plotted against calculated cell concentration based on serial dilutions on a log scale. All data points (5 per concentration) were plotted. A regression line was fitted to all data points *>*10^8^ cells/mL. The left-to-right order of samples in (a) matches the left-to-right order of data points in (b). (c) Calculated sperm concentration in a blind trial correlated closely with known concentration. A point falling exactly on the y = x line would indicate an exact match between the ‘known’ dilution of the sample and the value produced by calculations with data from the CMAS. A best fit regression line was fitted to all concentrations ≥10^8^ cells/mL. The points corresponding to samples at 5 × 10^7^ cells/mL appeared on the plot directly below the samples at 1 × 10^8^ cells/mL, suggesting that the CMAS may have reached the lower bound of its sensitivity at 1 × 10^8^ cells/mL.

The ten serially diluted catfish sperm samples (Fig. 5a) produced sensor voltages (i.e., the output voltages from the operational amplifiers) that were plotted against their concentrations (Fig. 5b). A linear regression was fitted for all concentration values above 10^8^ cells/mL. The equation of the best fit regression line for this concentration range was found to be

$$y=-1.9658\log_{10}x+19.702,\;r^{2}=0.9937$$

as compared to the best fit line with all points counted,

$$y=-1.3087\log_{10}x+13.822,\;r^{2}=0.9103\;.$$

In the blind concentration estimation trial, the cell concentration corresponding to each optical signal measurement was estimated from the inverse of the equation of the line derived in Fig. 5b, i.e.,

$$\text{cellconcentration}\;[\;\text{cells}\;/\text{mL}]{\text{=10}}^{\left[\text{10}-(\text{0}.\text{508}\times \;\text{sensor}\;\text{voltage}\;)\right]}$$

The records from the blind trial were revealed, and the ‘known’ sperm concentrations were plotted against those calculated from the CMAS data (Fig. 5c). The known and calculated concentrations were found to correlate closely above a cell concentration of 10^8^ cells/mL, but samples below that concentration threshold (5 × 10^7^ cells/mL) were not clearly distinguishable from 1 × 10^8^ cells/mL.

#### 3.2.2. Unicellular algal samples: concentration estimation and automatic dilution

The algal absorbance profile as measured by the Nanodrop 1000 (Suppl. Fig. E.2) showed no prominent peaks but was maximal at 229 nm, then declined gradually into the visible spectrum, with a near-constant absorbance at approximately 10% of maximum from 550 to 675 nm, and further decreased at increasing wavelength.

**Fig. 6.**
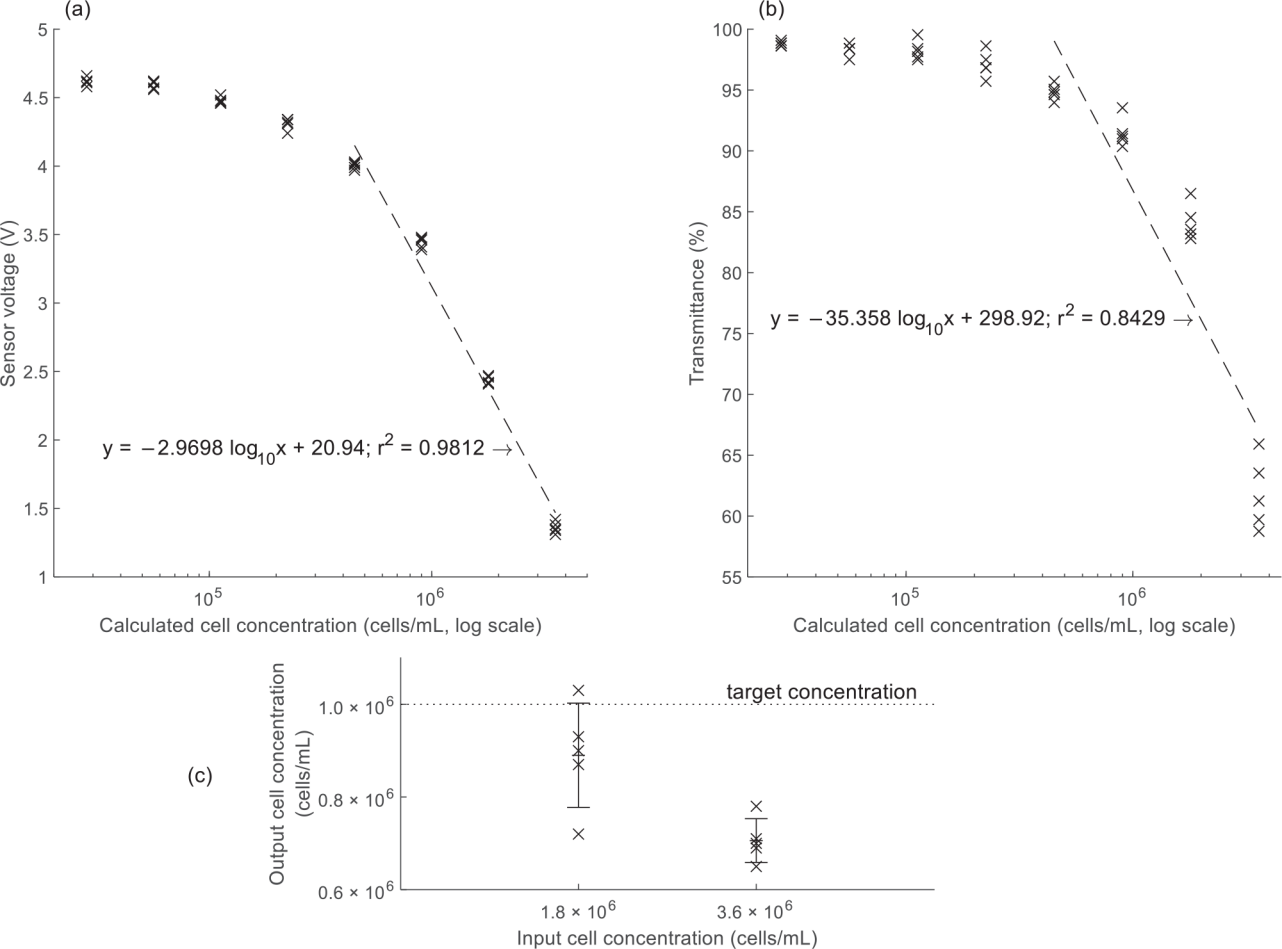
(a) Optical signals from algal samples evaluated on the CMAS were fitted by linear regression. The fitted equation allowed the CMAS to calculate the cell concentration of samples, with the aim of finding a suitable dilution ratio for the sample and adjusting to a target concentration. (b) The transmittance of the same algal samples as in panel a, evaluated on a Nanodrop 1000 spectrophotometer (*λ* = 625 nm). The linear regression served to indicate that the transmittance data obtained with the CMAS can be matched to a simple linear fit more readily than the Nanodrop data. Qualitatively, the sensitivity and technical replicability achieved with the CMAS sensor compare favorably to the Nanodrop in the tested range. (c) Results of automatic dilutions by the CMAS of algal samples. The target concentration from both starting concentrations was 1.0 × 10^6^ cells/mL. The symbol “ × ” indicates individual data points; whiskers indicate one standard deviation from the mean.

The CMAS optical signals from the serially diluted algal samples were plotted (Fig. 6a) and the best fit regression line for samples of concentration *>*(3 × 10^5^ ) cells/mL was determined to be

$$y=-2.9698\log_{10}x+20.94,\;r^{2}=0.9812$$

as compared to the best fit line for all data points,

$$y=-1.4644\log_{10}x+11.713,\;r^{2}=0.8285\;.$$

The CMAS was configured to calculate cell concentrations according to the function inverse to the best fit line for cell concentrations *>*(3 × 10^5^ ) cells/mL:

$$\text{cellconcentration}\;[\text{cells}/\text{mL}]=10^{\left[7.05-(0.337\times \;\text{sensorvoltage}\;)\right]}$$

The optical absorbances of the eight serial dilutions of algal samples, as measured on the Nanodrop 1000, were plotted against cell concentration. All samples of concentration *>*(3 × 10^5^ ) cells/mL were fitted to a regression line (Fig. 6b), determined to be

$$y=-35.358\log_{10}x+298.92,r^{2}=0.8429\;.$$

Algal cell concentrations resulting from automatic dilution by the CMAS were plotted (Fig. 6c). The target concentration in all cases was 1 × 10^6^ cells/mL. Starting from the lower initial concentration (1.8 × 10^6^ cells/mL), the CMAS produced an adjusted concentration of 8.9 (SD, 1.1) × 10^5^ cells/mL, representing a precision of ±12.6% and a mean of 89% of the target concentration. Starting from the higher initial concentration (3.6 × 10^6^ cells/mL), the CMAS produced an adjusted concentration of 7.1 (SD, 0.5) × 10^5^ cells/mL, representing a precision of ±6.7% and a mean of 71% of the target concentration.

## 4. Discussion

### 4.1. Fabrication of CMAS prototypes

The CMAS was designed with flexibility of open fabrication methods in mind. Certain components, such as the peristaltic pumps, can be fabricated by printing with FFF [28] (thermoplastic) or SLA/MSLA [35] (resin) 3-D printers, or by machining from stock plastic or metal. Others, such as the frame, can be 3-D printed, laser-cut, or cut and drilled with traditional machine tools such as a bandsaw and drill press. Providing the design files in multiple, flexible formats (i.e. .f3d/.f3z, .stl, and .dxf) also allows for fabrication with a wide variety of technologies.

The case and 3-D printed components of the CMAS were designed in Autodesk Fusion 360 with best practices for parametric computer-aided design in mind, which increased the ease of later changes such as enlargement of the case from the second to the third prototype. A parametric .f3d file to allow the fabrication of such cases with arbitrary dimensions is available from the authors on request.

### 4.2. Evaluation of the performance of the CMAS with biological samples

The optical module of the CMAS was shown to be sensitive to sperm cell concentrations in a range from 1 × 10^8^ cells/mL to 4 × 10^9^ cells/mL. This corresponded to a useful range in the workflow for cryopreservation of sperm of fishes of biomedical and aquacultural interest [20,21,36]. Of 60 channel catfish evaluated at the AGGRC in 2021, only three had a pre-dilution sperm concentration exceeding 5 × 10^9^ cells/mL. The nonlinearity of signals from samples below 10^8^ cells/mL was similar to that seen in previous work on photometry of oyster sperm samples [37]. In the context of this catfish sperm evaluation and freezing work, the target concentration for cryopreservation was 1 × 10^9^ cells/mL [20] after addition of extender medium and cryoprotective agents. The CMAS therefore was found to cover a cell concentration range that would allow measurement of sperm concentration within a practical range throughout the collection, evaluation, and concentration adjustment workflow for sperm of the channel catfish.

The overlay of the algal absorbance distribution with the LED emission spectra showed that the algae had no sharp absorbance peak in the visible light spectrum. This suggests that the three colors of light from the LED may allow for similar optical sensitivity in the CMAS. However, algal absorbance is greater at shorter wavelengths; therefore, if increased sensitivity is necessary in future work, the blue LED channel may be a better option than the red channel.

As was true for the catfish sperm samples, the algal samples allowed for a close linear regression fit only in a limited range of concentration (from 4.5 × 10^5^ to 3.6 × 10^6^ cells/mL). Cryopreservation protocols have not yet been standardized for these algae to ensure sufficient cells to reliably initiate new algal cultures; therefore, it is not possible to comment on whether this range is directly relevant to cryopreservation. However, the CMAS may at least be of general use in adjusting the concentration of algal samples drawn directly from a dense culture, as was the case for the sample used here.

The optical sensitivity of the CMAS in detecting cell concentration compared favorably to that of the Nanodrop 1000. The regression lines obtained for the CMAS (*r*^2^ = 0.9812) and the Nanodrop 1000 (*r*^2^ = 0.8429) for the same range of concentrations indicated that the CMAS produced a more closely linear response in the measured region. From the perspective of programming a microcontroller, performing calculations directly with the optical sensor voltage without converting to absorbance was simpler with a satisfactory result, as shown by the goodness-of-fit of the linear regressions for both catfish sperm and algae.

Evaluation of the concentration adjustment function showed that the components of the CMAS can work together to estimate and adjust the cell concentration of a sample. The trials with algal samples demonstrated reasonable precision in production of adjusted concentrations (±6.7% and ±12.6%; Fig. 6c) but lower accuracy (the trials produced mean cell concentrations that were 71% and 89% of the target concentration, i.e., 29% and 11% below the target). This may have arisen due to algae sticking to the inside of tubing, undetected variation in the flow rate produced by the peristaltic pumps, or error associated with the measurement of the concentration of the sample to be diluted. If in future trials it is found that the CMAS consistently dilutes to a lower concentration than the target, it may be necessary to compensate for this tendency directly in software. It is important to note that measurement accuracy within this range is fully acceptable for practical application, in which consistent adjustment of cell concentration to within half an order of magnitude of the target concentration is a reasonable standard in high throughput processing. This represents a substantial improvement to the current situation, where most aquatic cryopreservation is performed without measuring and adjusting cell concentration.

### 4.3. Benefits in the laboratory

The benefits of the CMAS to laboratory workflow will depend in part on the volume and number of samples to be processed. According to existing workflow modeling [38], estimation of the motility and concentration of a catfish sperm sample and adjustment to a target concentration requires on average 288 s (i.e., 12.5 samples/h) for an experienced technician. This manual throughput depends strongly on the number of samples to be processed, but it is only slightly affected by the volume of each sample; the timing is dominated by assessment and calculation.

Processing time with the CMAS, in contrast, depends on number of samples and on sample volume. A tubing replacement, if necessary to prevent cross-contamination between samples, required approximately 30 s, and priming the tubing of the CMAS with sample required ~20 s (priming was verified visually). If the peristaltic pump that draws sample could be run at maximum flow rate (1.1 mL/min), processing of a single sample would require (50 + 55•*V*) s, where *V* is the volume of the sample in mL. The processing time for a 4.3-mL sample (final adjusted volume 8.6 mL) on the CMAS (287 s) would be therefore similar to the equivalent manual processing time (288 s). The CMAS would result in a time savings for samples *<*4.3 mL, but an increase in processing time for samples *>*4.3 mL. The comparison is not exact because the manual process includes estimation of motility, a function not provided by the CMAS. However, this comparison serves as a starting point to estimate the time savings provided; it could be refined based on laboratory-specific needs and practices.

### 4.4. Future improvements

In future work, the CMAS could be improved in several respects. 1) To adequately compress the chosen tubing in the peristaltic pumps, substantial spring force was needed. A choice of softer tubing could permit the use of weaker springs and could improve the pump efficiency. For example, replacement of tubing in the pumps would be made easier. However, it would also require remeasurement of flow rates. 2) The electronics, which are currently laid out on soldered breadboard, could be adapted to a custom printed circuit board design. An efficient surface-mount design would greatly reduce the physical footprint of the electronics and allow for a more compact form factor [39]. 3) The casing could also be better sealed to prevent the incursion of fluids, which can be a common risk in a laboratory or hatchery environment. These improvements would improve the utility, safety, and reliability of the device. 4) Flow rates could be increased with different choices of motor, control circuitry, or tubing diameter, thereby reducing processing time and creating a time savings over manual processing of large volumes of sample. 5) A more sophisticated nonlinear or multi-section linear fit model could improve the ability of the CMAS to estimate cell concentration from a wider range of concentrations. 6) Light-emitting diodes producing infrared light or light frequencies apart from the red, green, and blue currently available could be integrated into the optics to permit interrogation of a wider range of sample absorbance profiles.

There is provision in the current design of the CMAS to mount as many as three pumping sub-modules and five optical evaluation sub-modules total. This could allow the dilution of an evaluated sample (pumped by one pumping sub-module) with a first-stage extender (pumped by a second sub-module), followed by a cryoprotectant solution (pumped by a third sub-module). All three fluids could be measured before mixing, each by its own optical evaluation sub-module; another evaluation could be made after the mixing of the sample and the extender; and a final evaluation could be made at the end of the workflow to verify the final concentration achieved. At that point, samples would be ready to be packaged for freezing.

The frame of the pump in early prototypes proved vulnerable to material fatigue and failure during use over several days. This was likely due in part to narrow features used in the frame, stress risers (i.e., sharp inside corners) in the design, and the constant spring force exerted on the frame. These early failures were largely rectified in later versions by designing and printing a more robust frame, with thicker geometric features and 100%-density 3-D printed infill. Future work could test and further improve the robustness of the frame to prevent fatigue over weeks and months of use at points placed under frequent or constant stress.

The CMAS could be integrated with other devices to improve the efficiency of cryopreservation workflows for aquatic germplasm: for example, an open hardware version of a filler–sealer device for French straws, which are widely used as containers for animal germplasm [40]. This device would be an obvious candidate for integration as a processing step immediately downstream from the CMAS. It could be a productive technology development project to connect the fluidic output of the CMAS to the fluidic input of the French straw filler–sealer and coordinate the action of the two devices.

## 5. Conclusions

Aquatic species are incredibly diverse with numerous species groups, industries, applications, and global user communities that present a wide range of specific needs. Open hardware offers many approaches to address the needs for aquatic germplasm preservation, but current options are mostly based on 3-D printed parts. Therefore, our goal was to combine multiple open technologies and fabrication methods into a composite functioning device that addressed significant barriers to repository development that can be specifically adapted to the needs of the many diverse aquatic user communities. The CMAS was intended as high-level source material for others to advance and adapt, not to be a final product for widespread off-the-shelf use. This is an example of creating a new category of device – not a final device according to traditional engineering thinking. Open-source hardware depends on community interaction and improvements – not traditional products that move from a single (often proprietary) source to the customer.

Thus the CMAS was designed and fabricated as an open-technology platform that can serve as a starting point for further refinement and for future technology development. This device provides an opportunity to increase the reach of open technologies in the aquatic germplasm community [29,41]. It is a design amenable to open, distributed fabrication by 3-D printing, laser cutting, and hand-soldering. It was demonstrated in this work that such a device can effectively evaluate cell concentration in fluidic samples and dilute them to a target concentration, to a precision of ±12.6% and ±6.7% in two trials, with a final cell concentration 89% and 71% of the target concentration. These activities are essential but typically neglected features of the cryopreservation process [12,42], and they are targets for improvements in efficiency and throughput such as those offered by the CMAS. Encouraging laboratories to engage in the open-technology community [30] and to fabricate their own versions of open-technology tools and devices such as the CMAS can improve the quality of germplasm samples, increase cryopreservation throughput through distributed processing, and strengthen aquatic germplasm repositories globally.

## Supplementary Material

1
