# Supplementary material for: An automated modular open-technology device to measure and adjust concentration of aquatic sperm samples for cryopreservation
Source: SLAS Technol. Author manuscript; Available in PMC 2023 Feb 27. (PMC9969519; doi:10.1016/j.slast.2022.11.002)
Supplement: 1 [file NIHMS1871913-supplement-1.zip › Supplementary material - Appendices.pdf]

SUPPLEMENTARY MATERIAL

Journal: SLAS Technology

Article: An Automated Modular Open-Technology Device to Measure and Adjust Concentration of Aquatic Sperm Samples for Cryopreservation

Nikolas Zuchowicz[a], Yue Liu[a,b], W. Todd Monroe[b], Terrence R. Tiersch[a]

Author affiliation:

[a] Aquatic Germplasm and Genetic Resources Center, School of Renewable Natural Resources, Louisiana State University Agricultural Center, Baton Rouge, LA, USA

[b] Department of Biological and Agricultural Engineering, Louisiana State University & LSU Agricultural Center, Baton Rouge, LA, USA

Supplementary Material A: Guide to Fabrication and Assembly of the Concentration Measurement and Adjustment System

Overview

The CMAS was fabricated using three technologies: 3-D printing, laser cutting, and soldering. It is expected that the maker will have some familiarity with each of these or will be willing to learn independently. Concise fabrication instructions are provided here with the more experienced maker or developer in mind. Many tutorials are available online that introduce these fabrication technologies. Three starting points are suggested here.

3-D printing: hubs.com/knowledge-base/introduction-fdm-3d-printing
Laser cutting: hubs.com/blog/laser-cutting
Soldering: makerspaces.com/how-to-solder

3-D printing of .stl files

3-D printing files are provided in .stl (‘stereolithography’) format, a simple mesh representation of 3-D geometry made up entirely of triangular faces. These files contain no information specific to one 3-D printer or material, nor do they explicitly include dimensions (although they are implicitly measured in mm, which is the usual unit for 3-D printing software). The maker must use software called a *slicer* to convert the .stl files into a format that the 3-D printer will be able to use.

The files provided herein (Table A.1) were processed in Cura slicing software 4.9.0 (Ultimaker, Utrecht, Netherlands) and 3-D printed with polylactic acid (PLA) thermoplastic (eSun, Shenzhen, China, esun3d.net) on an Ultimaker 3 Extended (Ultimaker) 3-D printer (Figure A.1). However, it should be possible to produce the necessary parts on any common 3-D printer. Note that the optical enclosures should be printed in black or another dark, opaque filament to help exclude light.

Table A.1. 3-D printed components. These parts can be printed in any color of filament except where otherwise noted.

Component	Quantity	Note	Image
Card slot	1		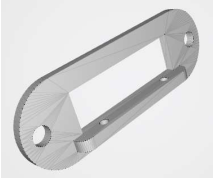
Encoder knob	1		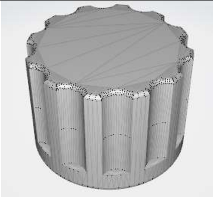

(table cont’d.)

Component	Quantity	Note	Image
Encoder slot	1		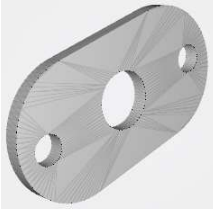
Frame brace	2		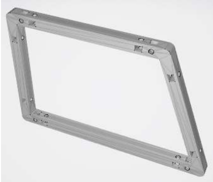
Frame foot	4		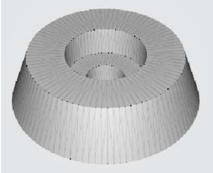
Frame handle	2		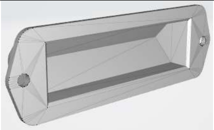
Optics base	7	5 should be printed in black (for opacity); the other 2 can be any color	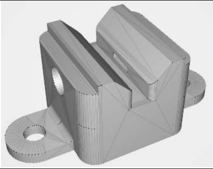
Optics cap	5	1 printed in black for each populated optics group	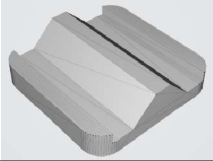
Pump base	3		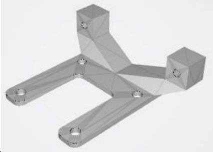
Pump motor spacer	3		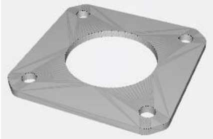
Pump rider	3		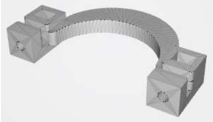
Pump rotor cap plate	3		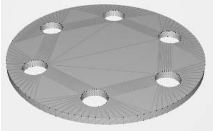

(table cont'd.)

Component	Quantity	Note	Image
Pump rotor half	6		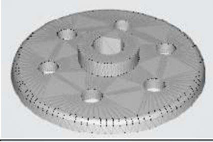
Tube holder	4		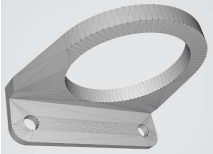
Tube adapter insert 1.5-mL	4	Only print if needed	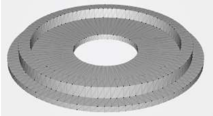
Tube adapter insert 15-mL	4	Only print if needed	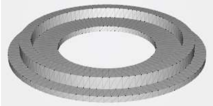
Tubing clamp	3	Keeping several spares is recommended to help with changing out tubing	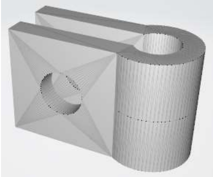

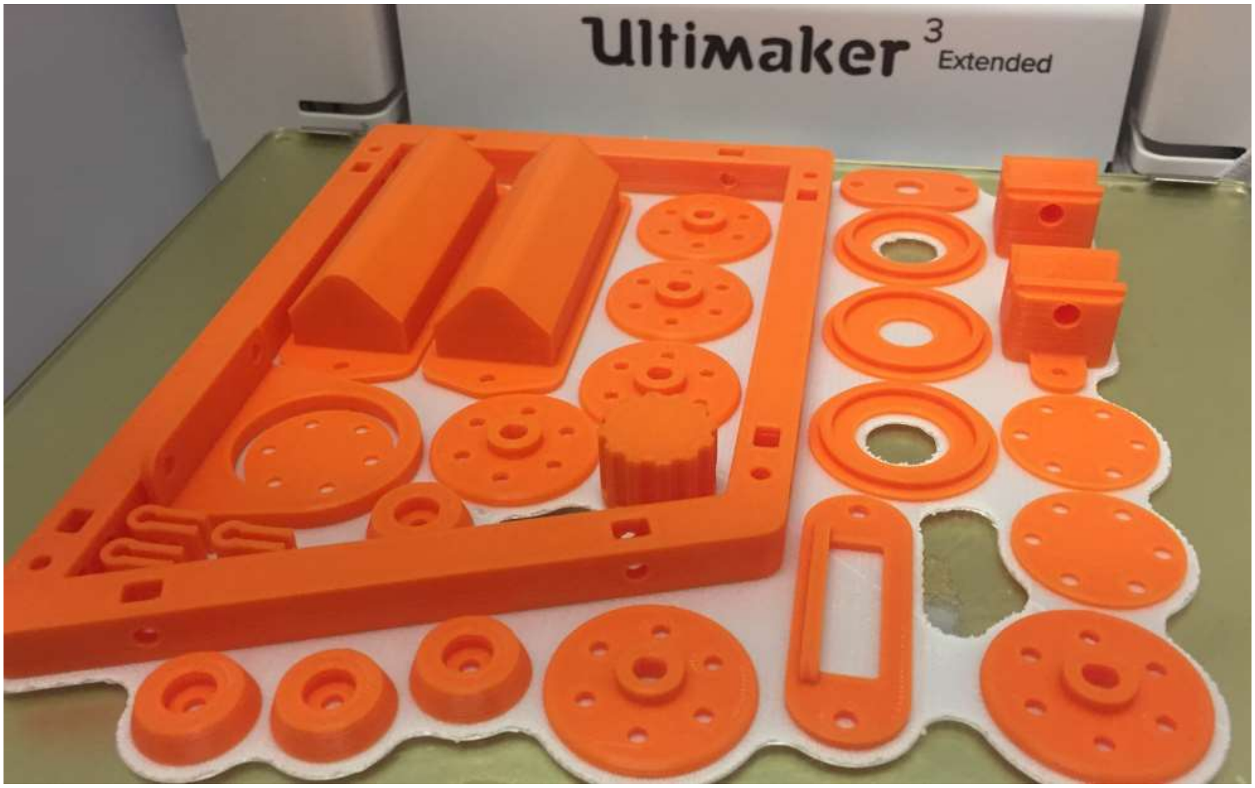

Figure A.1. An assortment of CMAS parts printed on the Ultimaker 3 Extended 3-D printer. The white support material is PolyMaker PolySupport filament; the orange is eSun polylactic acid.

Laser cutting the body panels

The body panels of the CMAS are designed to be cut from 1/4” (6 mm) acrylic (PMMA). The exact method for cutting will vary according to the laser used. This work used a Muse 1064-nm CO_2 laser cutter (Full Spectrum Laser, Las Vegas, Nevada, USA) with a 12 × 20” cutting bed, which allowed cutting of multiple panels from a single sheet of material. The panels were cut completely through with three passes of the laser at 50% speed, 100% power, and 100% current (as set in the custom firmware that runs the laser cutter). As an aid in planning and splitting material for cutting, the dimensions and quantities of each panel are summarized in Table A.2.

Table A.2. Body panels to cut. The given dimensions are the maximum dimensions of each panel, *i.e.* the dimensions of the rectangular bounding box containing the panel.

Panel	Dimensions (mm)	Dimensions (in)	Quantity	Image
Front	298.0 × 123.4	11.74 × 4.86	1	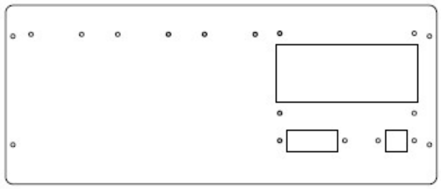
Top	298.0 × 152.2	11.74 × 6.00	1	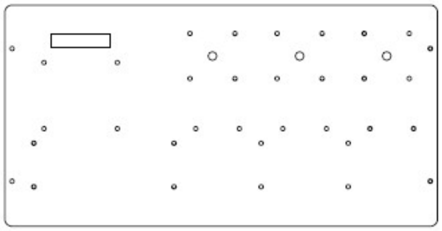
Back	298.0 × 118.0	11.74 × 4.65	1	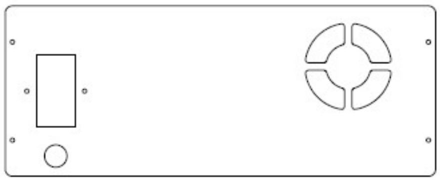
Bottom	298.0 × 195.2	11.74 × 7.69	1	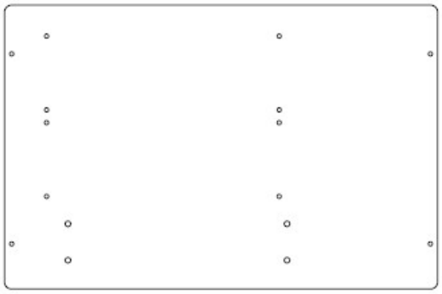
Side	195.2 × 130.0	7.69 × 5.12	2	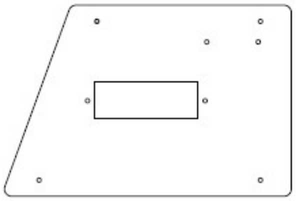

Other fabrication methods are possible, although more laborious, without access to a laser cutter. For example, the panels could be cut with a bandsaw and a drill press. In this case, the provided patterns (in .dxf format) could be printed out on plain office paper and glued with spray adhesive to the acrylic. The cuts and holes could be made following the printed patterns. Nor must the panel material be acrylic: fiberboard or thin plywood should serve equally well.

Assembling the electronics

Fabricating the electronics requires several skills: reading circuit diagrams, identifying electronic components, soldering, and crimping wire connectors.

Layout

A pin-by-pin layout of the breadboards is not provided here, but full circuit diagrams are provided in Appendix C. A possible layout is provided (Figure A.2) to guide planning of breadboard space.

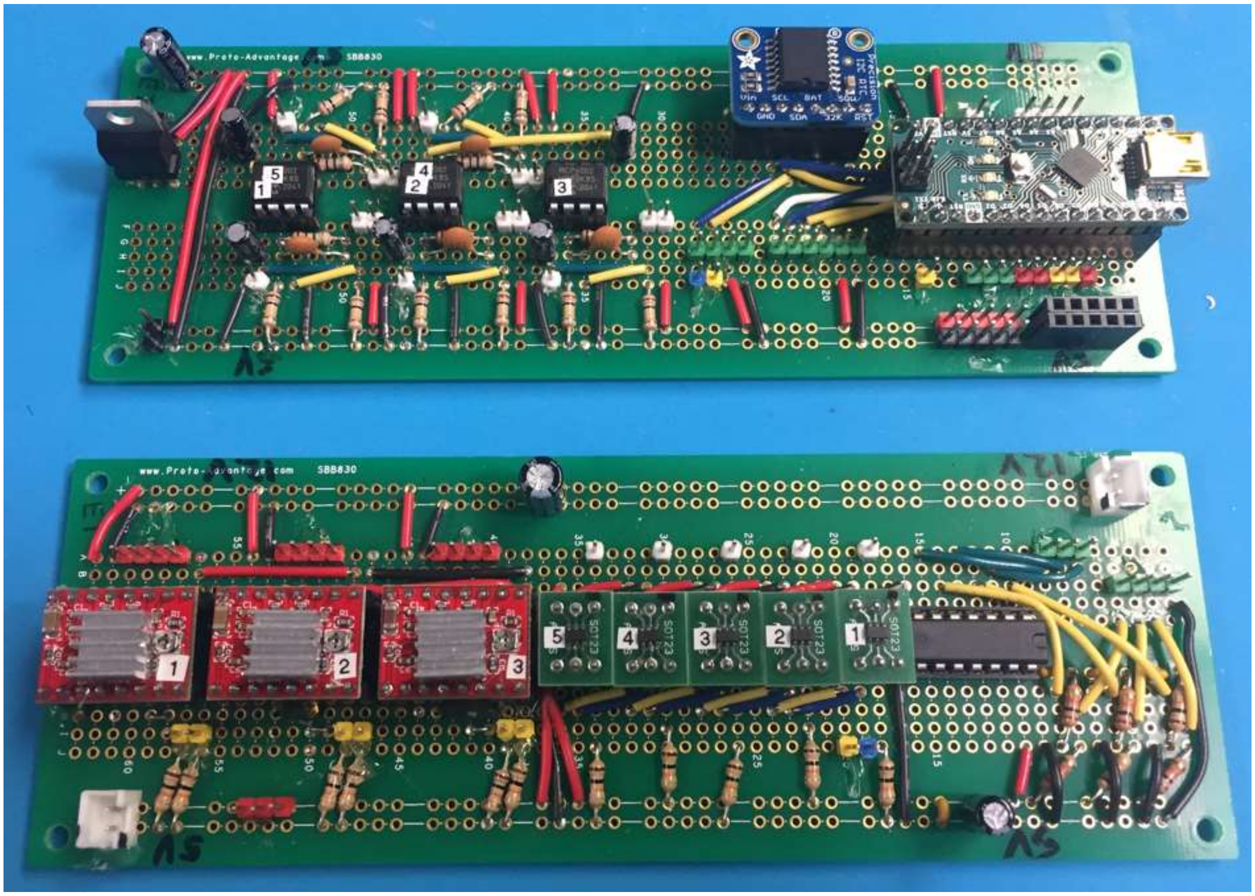

Figure A.2. The two main breadboards of the CMAS.

Soldering

Most soldering for the CMAS is conventional through-hole soldering. However, the DAC ICs are only available in a surface-mount package (Figure A.3). It was therefore necessary to

solder the DACs to 6-pin 0.1” SOT-23 adapter boards, which matched the pitch of standard through-hole breadboards. This can be done by hand with drag soldering or a similar technique. This is made easier by the careful selection of a soldering tip (a drag soldering tip is suggested in the Bill of Materials, Appendix B) and a generous application of flux to prevent solder bridging.

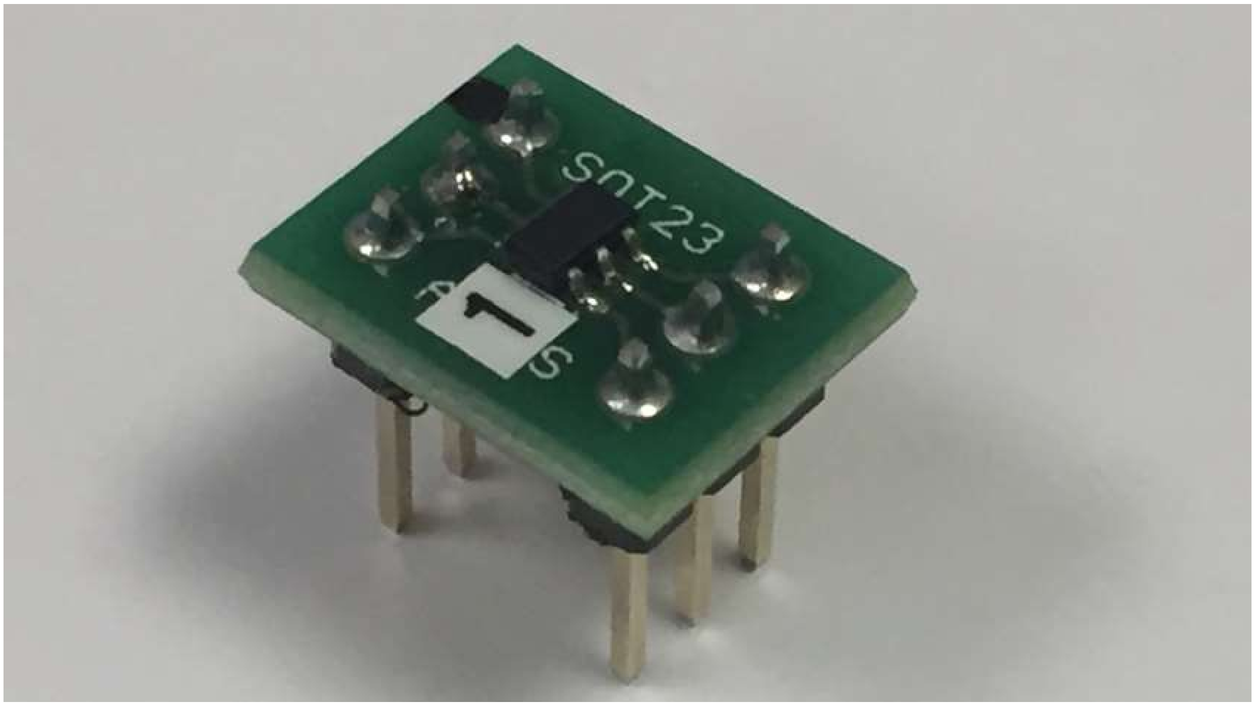

Figure A.3. A DAC IC in a SOT-23 package soldered to an adapter board.

Wiring and labeling

This design can include a large number of wires to connect power and data to the various components. For assembly and later troubleshooting, it is best to consistently and cleanly label each wire as it is cut and crimped. One possible approach to this labelling is suggested in Ch. 5. The exact wiring configuration (Figure A.4) will be up to the individual maker and the available wiring supplies, although some Dupont crimp terminals and a crimper are suggested in the Bill of Materials.

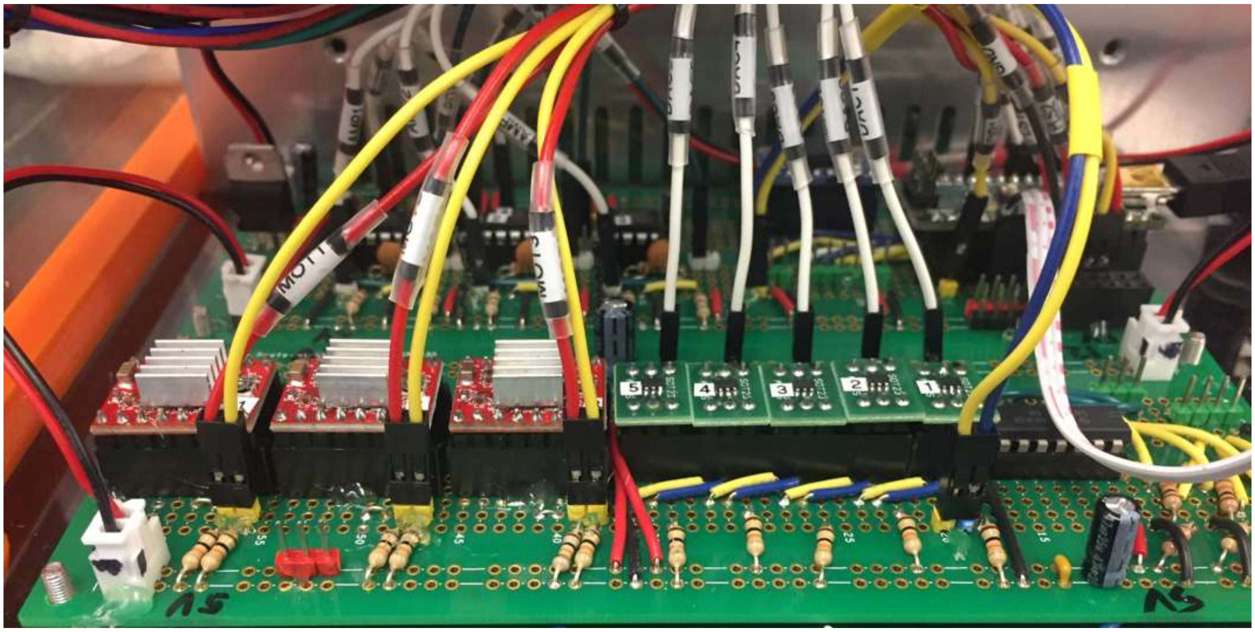

Figure A.4. An example of wiring in the CMAS.

Assembling the frame

It is easiest to assemble the frame by installing the power supply and frame braces onto the bottom panel first (Figure A.5a). Doing so before installing the back panel allows access to the power supply distribution terminals for connection of external power (A.5b).

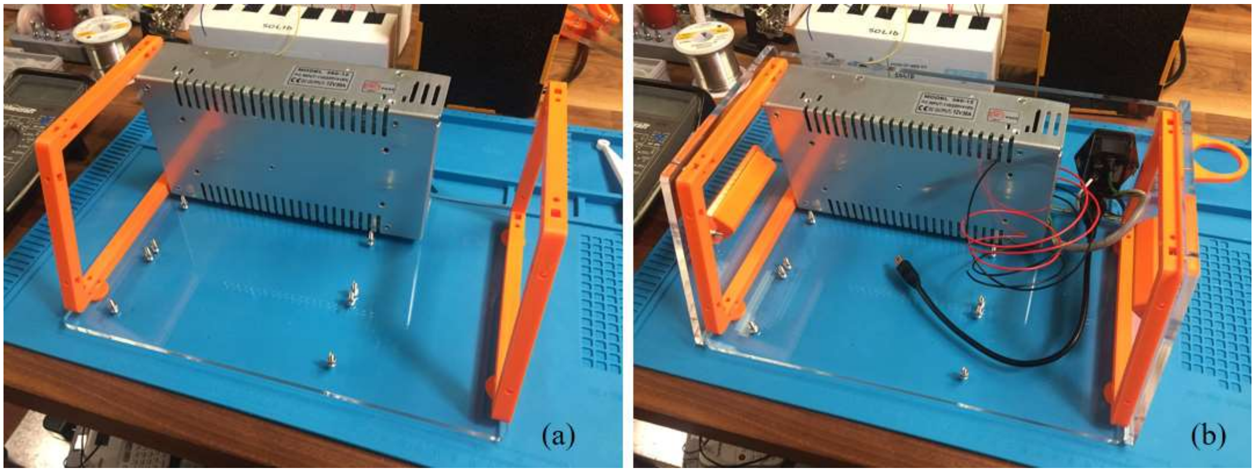

Figure A.5. Assembling the frame. (a) Installation of the power supply and frame braces on the bottom panel. (b) Connection of external power, and installation of the back and side panels.

The top and front panels, including the pumps, optics, and user interface components, are meant to be easily removed (Figure A.6), and are best installed last (Figure A.7).

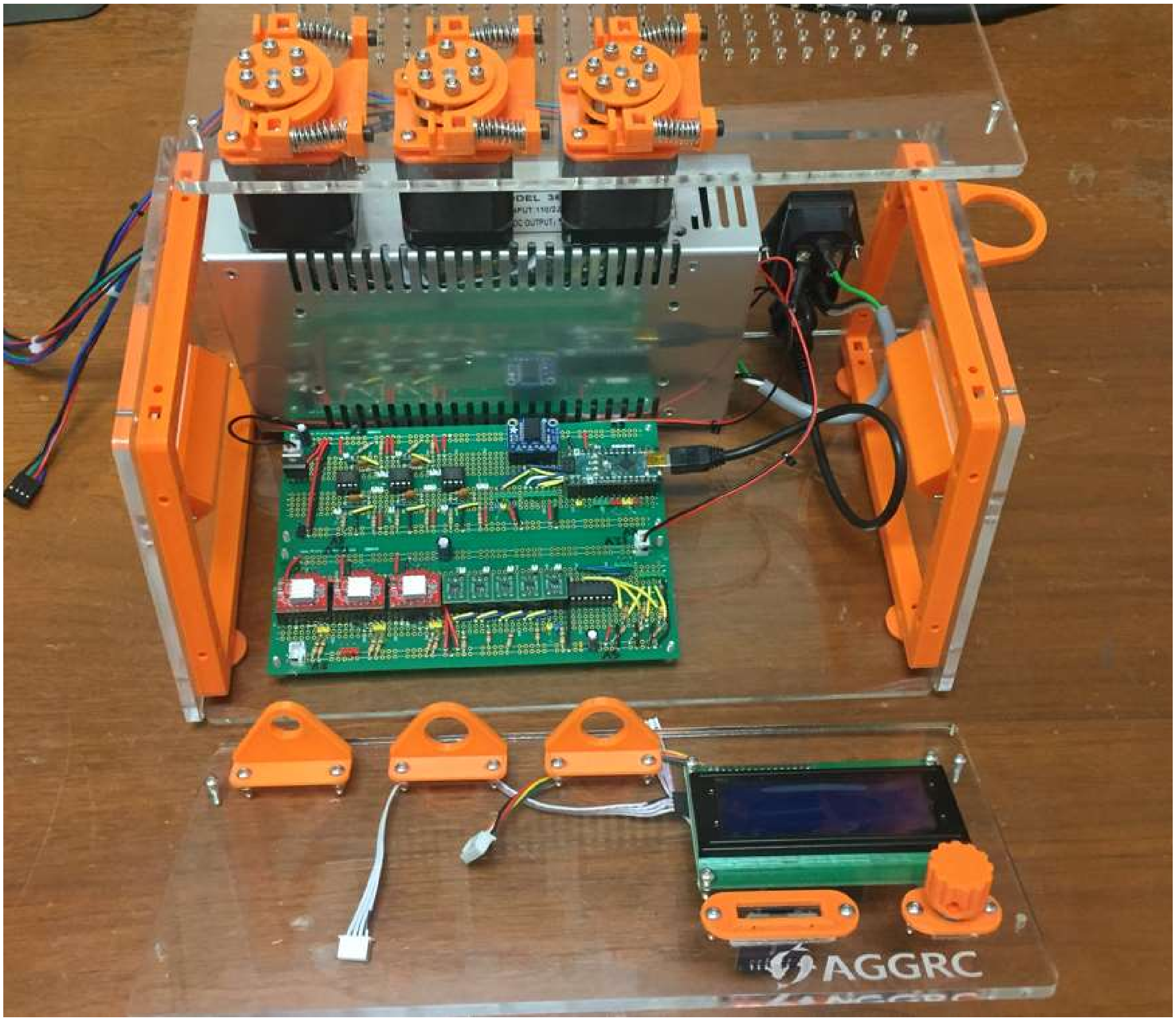

Figure A.6. Assembly of the top and front panels. Installation of the breadboards onto the bottom panel.

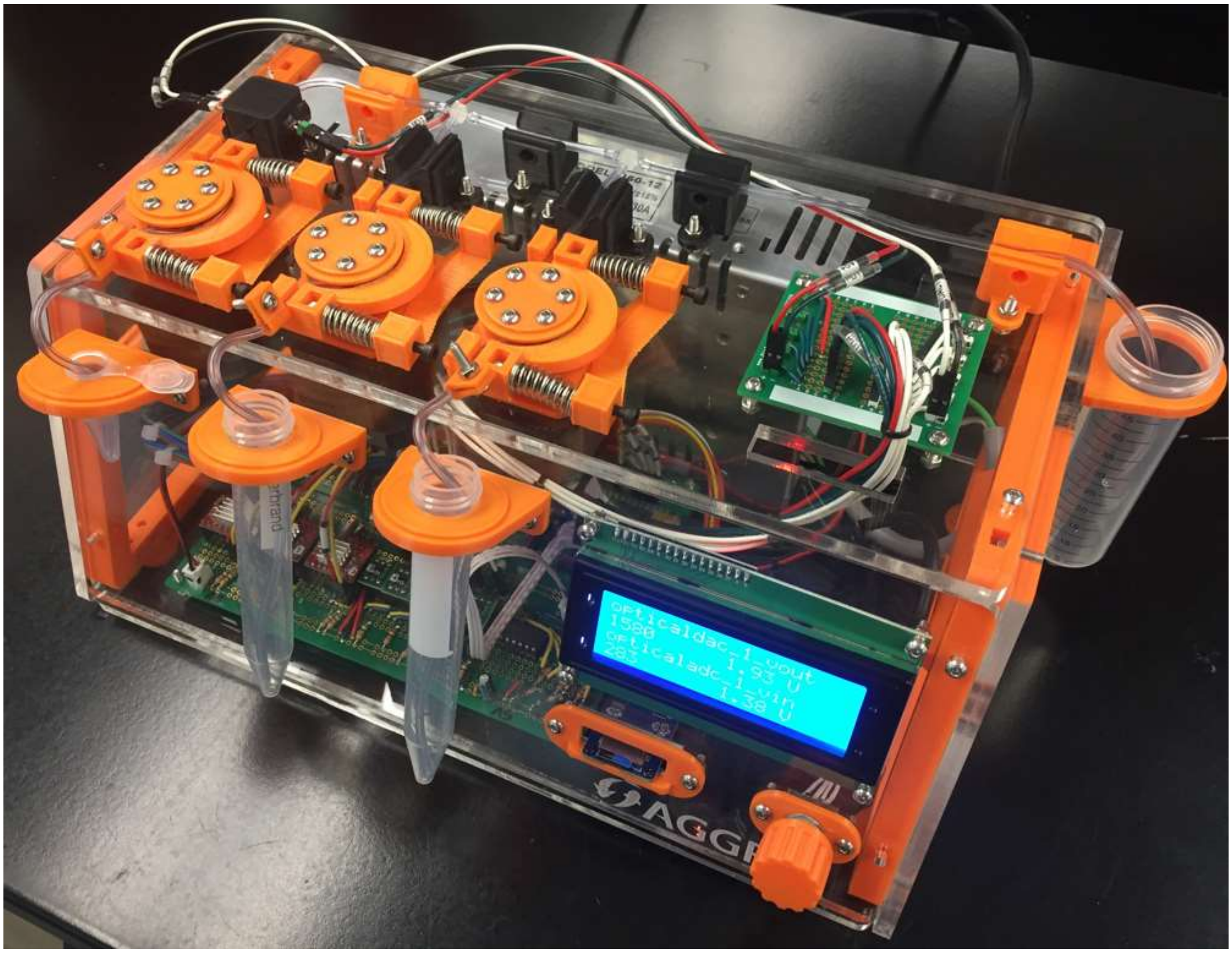

Figure A.7. The assembled CMAS.

Supplementary Material B: CMAS Bill of Materials

Quantities for certain bulk purchases are given as follows: “1 (10)” means the item is available as a package of 10, but only 1 is used in the project. The cost given is for the full package of 10. “3 (3)” means that 3 are required, and the cost given is for a pack of 3. Costs are estimates in US dollars as of 2021. No tax or shipping is included. “Ext.” is the extended cost, i.e., (cost × quantity). Many items, especially the tools, are listed only as suggestions and may be substituted with another brand. For example, the Ender 3 3-D printer is only one of many entry-level 3-D printers offered; likewise with the Hakko FX888D soldering station. Many items are available from other vendors; the links listed are provided for convenience.

Table B.1. Fabrication technologies and tools.

Item	Manufacturer	Comment	Link	Cost	Quantity	Ext.
Ender 3 3-D printer	Creality		amazon.com/dp/B07BR3F9N6	174.99	1	174.99
Muse CO_2 laser cutter	Full Spectrum Laser		fslaser.com/musecore/	3524.00	1	3524.00
Hakko FX888D soldering station	Hakko		amazon.com/dp/B00ANZRT4M	104.95	1	104.95
T18-D16 soldering chisel tip	Hakko	General-purpose tip	amazon.com/dp/B004OR9BV4	8.99	1	8.99
T18-CF15 soldering bevel tip	Hakko	For surface-mount components	amazon.com/dp/B004OR9BLO	9.99	1	9.99
Solder	Yi Lin		amazon.com/dp/B08RHMPJW3	8.99	1 roll	8.99
Flux pen	Chip Quik		amazon.com/dp/B07B53LNGX	7.95	1	7.95
Fine tweezers	Hakko	For surface-mount components	amazon.com/dp/B00FZPESTY	16.07	1	16.07
Solder wick	NTE Electronics		amazon.com/dp/B0195UVWJ8	9.74	1 roll	9.74
Flush cutters	Hakko		amazon.com/dp/B00FZPDG1K	7.47	1	7.47
Lighter	BIC	To heat shrink tubing	amazon.com/dp/B086R3LFBW	11.98	1 (10)	11.98

Item	Manufacturer	Comment	Link	Cost	Quantity	Ext.
Screwdrivers, metric hex set	Wiha	2.0 mm for M3 screws; 2.5 for M4 and M3 smooth shaft	amazon.com/dp/B000NZ5QE2	19.43	1 set	19.43
Needle nose pliers	Boosden		amazon.com/dp/B093K71F9F	13.99	1	13.99
Parallel-jaw pliers	Mazbot	Not required, but useful for gripping hex nuts	amazon.com/dp/B01FSV4PME	19.95	1	19.95
Wire strippers	Klein Tools	To strip wire insulation	amazon.com/dp/B0000302WS	11.99	1	11.99
Small zip ties	Laishuo	To keep wire bundles neat	amazon.com/dp/B075F78RX5	11.99	As needed (500)	11.99
Hot glue gun	Daikoye	Used as a soldering aid	amazon.com/dp/B095C32T5C	9.99	1	9.99
Label maker	Brother	To mark wires, (a pen and masking tape will work)	amazon.com/dp/B013DG2FNW	34.99	1	34.99
Wire bender	-	3-D printed	thingiverse.com/thing:1274814	-	1	-
Resistor lead bender	-	3-D printed	thingiverse.com/thing:4191881	-	1	-
IC lead straightener	-	3-D printed	thingiverse.com/thing:3121797	-	1	-

Table B.2. Hardware and raw fabrication materials.

Item	Manufacturer	Comment	Link	Cost	Quantity	Ext.
6-mm acrylic sheet	AZM Displays	Any color	amazon.com/dp/B07H9NQP8G	22.99	2	45.98
M3 machine screw 14 mm	iexcell		amazon.com/dp/B08H2HQ3VZ	5.99	105 (100)	11.98
M3 half thread socket cap machine screw 40 mm	iexcell	For peristaltic pump rider assemblies	amazon.com/dp/B076XKGNDB	10.99	6 (20)	10.99
M3 hex nut	Powlankou		amazon.com/dp/B082D6M295	5.99	75 (200)	5.99
M3 nylock hex nut	Eastlo		amazon.com/dp/B0865NLJNN	7.99	24 (100)	7.99
M4 machine screw 12 mm	Zayi	To attach power supply	amazon.com/dp/B084Q45BGR	6.91	4 (100)	6.91
Mini self-tap screws M2 6 mm	Helifouner	To secure microSD card reader	amazon.com/dp/B081DVZMHH	12.29	2 (60)	12.29
PLA 3-D printing filament	eSUN	Any color works, but optical enclosures are printed in black	amazon.com/dp/B01EKEMDA6	22.99	1 kg roll	22.99
PolySupport 3-D printing filament	Polymaker	Not needed, but useful for dual extrusion. Link is for 2.85 mm	amazon.com/dp/B07XP75M2Y	39.99	750 g roll	39.99
Ball bearings 3 × 9 × 5 mm	uxcell		amazon.com/dp/B082PQNVF7	8.89	18 (10)	17.78
Assorted springs	Neiko	Use 6 of (7/32 × 1 1/2”) and 6 of (23/64 × 1 3/8”)	amazon.com/dp/B000K7M36W	10.99	12 (20)	10.99
Plastic barbed tube fitting for	Unspecified		mcmaster.com/5116K183/	6.06	30 (10)	18.18

Item	Manufacturer	Comment	Link	Cost	Quantity	Ext.
food & beverage, 1/16” tube ID						
Continuous-flex soft plastic tubing, 1 mm ID 3 mm OD	Unspecified		mcmaster.com/3774N1/	31.50	50 ft (50 ft)	31.50

Table B.3. Electronic components.

Item	Manufacturer	Comment	Link	Cost	Quantity	Ext.
Arduino Nano	Arduino		amazon.com/dp/B0097AU5OU	19.80	1	19.80
Mini USB case-mount plate and extension	Kework		amazon.com/dp/B082J1BL6H	9.99	1	9.99
USB cable	Amazon Basics		amazon.com/dp/B00NH13S44	5.87	1	5.87
NEMA 17 stepper motor	StepperOnline		amazon.com/dp/B07LCK19D5	27.99	3 (3)	27.99
A4988 stepper motor driver module	Biqu		amazon.com/dp/B01FFGAKK8	10.99	3 (5)	10.99
Wall-socket-to-C14 power cable	Amazon Basics	Or regional equivalent	amazon.com/dp/B072BYGKZZ	8.99	1	8.99
C14 panel mount power socket	Muzhi		amazon.com/dp/B082ZFRV1B	8.49	1 (3)	8.49
12-V 30-A power supply	Bmouo		amazon.com/dp/B01EWG6YT8	17.98	1	17.98
I^2C 20×4 LCD module	SunFounder		amazon.com/dp/B01GPUMP9C	12.99	1	12.99

Item	Manufacturer	Comment	Link	Cost	Quantity	Ext.
Resistor 10 kΩ ¼ W	Stackpole Electronics		digikey.com/short/20n1pdq9	0.10	27	2.70
Resistor 100 kΩ ¼ W	Stackpole Electronics		digikey.com/short/0wnhpnrf	0.10	10	1.00
Solderable breadboard 400 point	Chip Quik		digikey.com/short/8cjwbjmt	3.99	1	3.99
Solderable breadboard 830 point	Chip Quik		digikey.com/short/rb8jt0q8	6.49	2	12.98
Pin header assortment, male & female	Cenrykay		amazon.com/dp/B07RRPYZCT	9.99	1 pack	9.99
Dupont connector assortment with crimping tool	JZK		amazon.com/dp/B07PCQ5VMV	26.89	1 pack	26.89
Solid 22 AWG wire, assorted colors	SparkFun Electronics	For use directly on soldered breadboard	digikey.com/short/885h54hn	16.95	1 pack	16.95
Stranded 22 AWG wire, assorted colors	SparkFun Electronics	For use in running between components	digikey.com/short/n8df2p9n	16.95	1 pack	16.95
JST-XH cables	Antrader		amazon.com/dp/B07DVFTGSL	7.99	As needed (50)	7.99
MCP6002 dual op-amp	Microchip Technology		digikey.com/short/8pn2j7t4	0.38	3	1.14
74HC14 hex inverting Schmitt trigger IC	Texas Instruments		digikey.com/short/hv82f71d	0.61	1	0.61

Item	Manufacturer	Comment	Link	Cost	Quantity	Ext.
Ceramic capacitor 100 pF	Vishay Beyschlag		digikey.com/short/rhntn9bv	0.26	5	1.30
Ceramic capacitor 0.1 µF	Kemet		digikey.com/short/t22bpcz9	0.23	6	1.38
Electrolytic capacitor 100 µF	Rubycon		digikey.com/short/2v0f9pvv	0.28	2	0.56
IC socket (14-pin)	TE Connectivity	May be swapped out for two 8-pin sockets	digikey.com/short/b9v5wzzn	0.27	1	0.27
IC socket (8-pin)	TE Connectivity		digikey.com/short/m9t4fm04	0.20	3	0.60
7805 linear voltage regulator	Texas Instruments		digikey.com/short/t5nqcw8t	0.89	1	0.89
Photodiode SFH 203 P	Osram USA		digikey.com/short/4rfq94dp	1.15	5	5.75
LED, tri-color QBL8RGB-60D0-2897	QT Brightek		digikey.com/short/zpt12zdd	1.05	5	5.25
DAC: A0 variant	Microchip Technology		Mouser: MCP4725A0T-E/CH	1.01	2	2.02
DAC: A1 variant	Microchip Technology		Mouser: MCP4725A1T-E/CH	1.01	2	2.02
DAC: A2 variant	Microchip Technology		Mouser: MCP4725A2T-E/CH	1.01	1	1.01
SOT-23 0.1” pitch adapter	Aries Electronics		Mouser: 535-LCQT-SOT23-6	4.49	5	22.45
Rotary encoder	Bourns		digikey.com/short/95tnfvv7	2.34	1	2.34
3-conductor mains power cable	-	Salvage from an old power cord	-	-	20 cm	-

Item	Manufacturer	Comment	Link	Cost	Quantity	Ext.
Shrink tubing assortment	Eventronic	To insulate wire connections	amazon.com/dp/B072PCQ2LW	6.49	As needed (1 pack)	6.49
microSD card breakout board	Stemedu		amazon.com/dp/B07MTTLF75	8.99	1 (5)	8.99
microSD card 32 GB	SanDisk		amazon.com/dp/B073JWXGNT	9.72	1	9.72
DS3231 real-time clock module	Adafruit		amazon.com/dp/B0718XYYTB	17.56	1	17.56
CR1220 battery	Eunicell	For real-time clock	amazon.com/dp/B0751YTGST	4.27	1 (10)	4.27

Table B.4. Total costs to build the CMAS.

B.1. Fabrication technologies and tools	4007.45
B.2. Hardware and raw fabrication materials	243.56
B.3. Electronic components	317.15
Total excluding fabrication technologies and tools (sum of tables B.2 and B.3)	**560.71**
GRAND TOTAL (sum of tables B.1, B.2, and B.3)	**4568.16**

Supplementary Material C: CMAS Circuit Diagrams

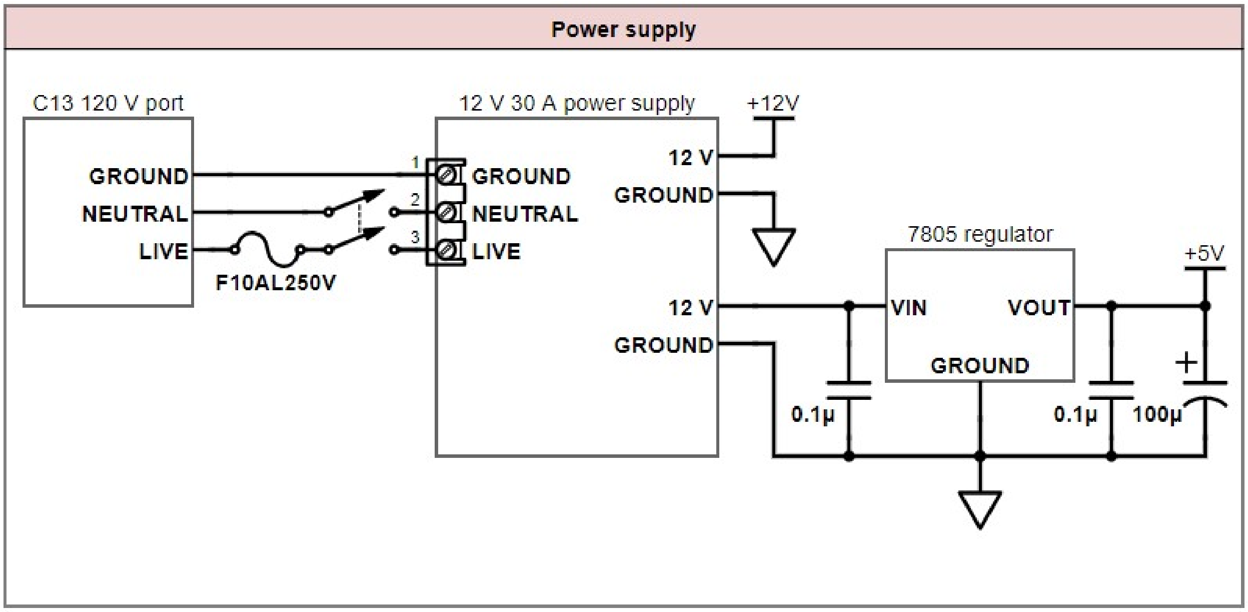

Figure C.1. Circuit diagram of the power supply.

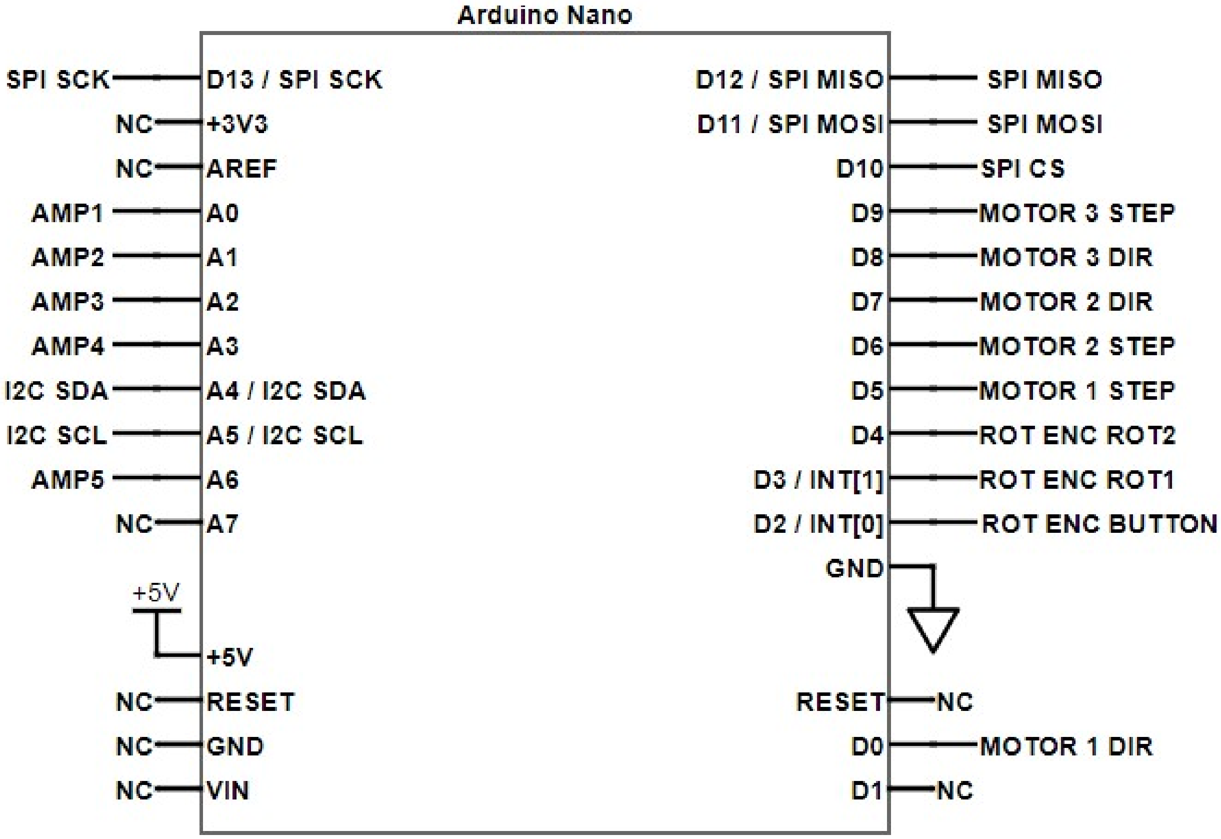

Figure C.2. Pinout of the Arduino Nano microcontroller platform. NC: no connection.

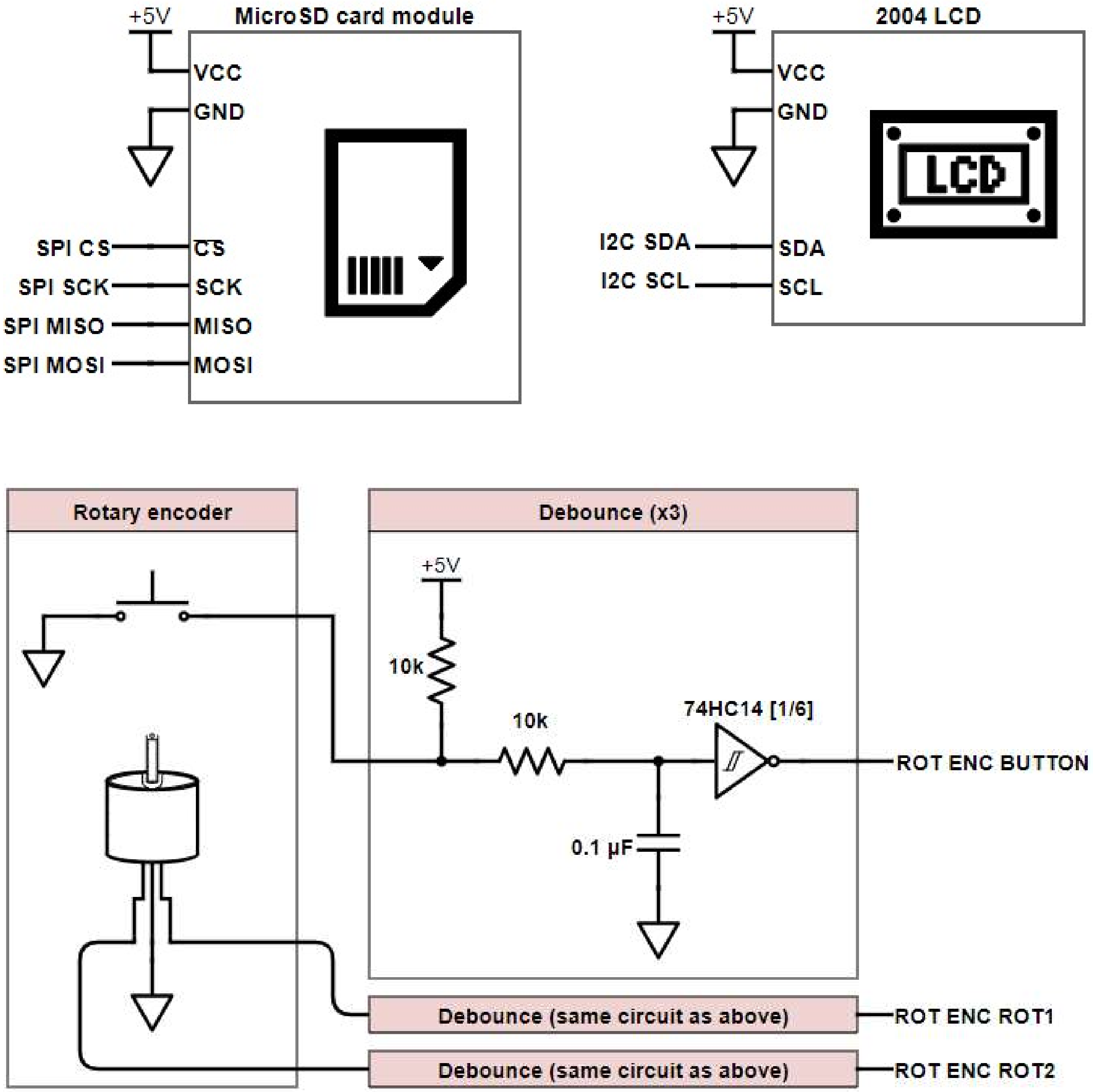

Figure C.3. Pinouts and circuit diagrams of the MicroSD card module, 2004 LCD module, and rotary encoder. One debounce circuit is needed for each rotary encoder output. The rotary encoder is a single electromechanical component incorporating a pushbutton (two pins) and a quadrature encoder (three pins). These two elements of the rotary encoder are shown separately in the “Rotary encoder” panel to clarify the electrical connections.

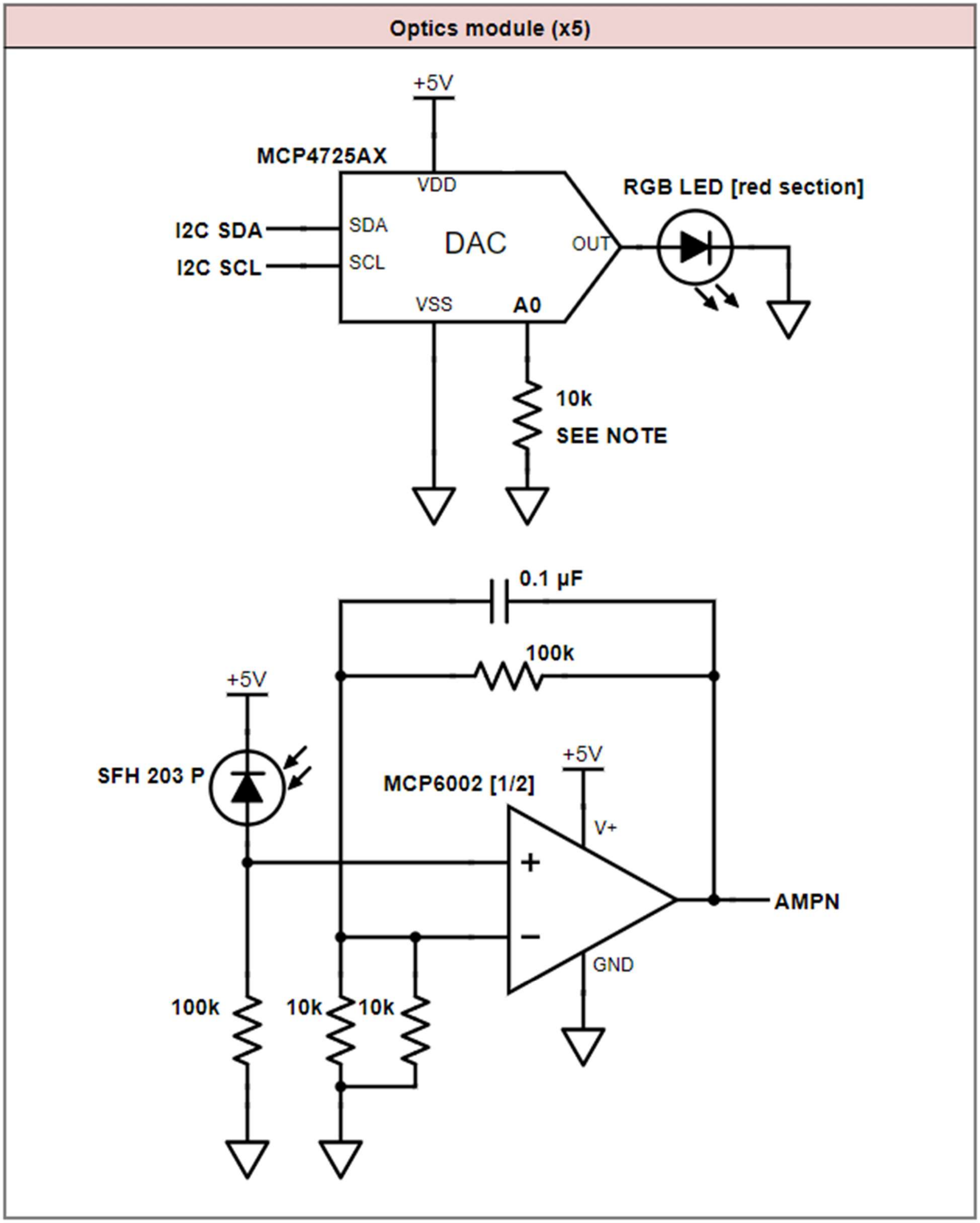

Figure C.4. Circuit diagram of an optics module. A module comprises an LED, the Digital–Analog Converter (DAC) supporting the LED, a photodiode, and the operational amplifier circuit supporting the photodiode. Up to five optics modules may be fitted to the CMAS. The five MCP4725 DAC ICs must each have a unique I^2C bus address to prevent conflict. This IC comes

in four variants, each of which has two possible addresses set by pin A0. The base address of each variant is different and is set at the factory. The variants differ only in their base address and in no other way. This design uses two of the A0 variant, two of the A1 variant, and one of the A2 variant. Pull pin A0 on each IC up to +5V via a 10 kΩ resistor or down to ground via a 10 kΩ resistor as follows. (Confusingly, variants A0, A1, and A2 all have a pin A0, which is the address set pin).
DAC IC 1: MCP4725A0 pull down (I^2C address 0x60)
DAC IC 2: MCP4725A0 pull up (I^2C address 0x61)
DAC IC 3: MCP4725A1 pull down (I^2C address 0x62)
DAC IC 4: MCP4725A1 pull up (I^2C address 0x63)
DAC IC 5: MCP4725A2 pull down (I^2C address 0x64)

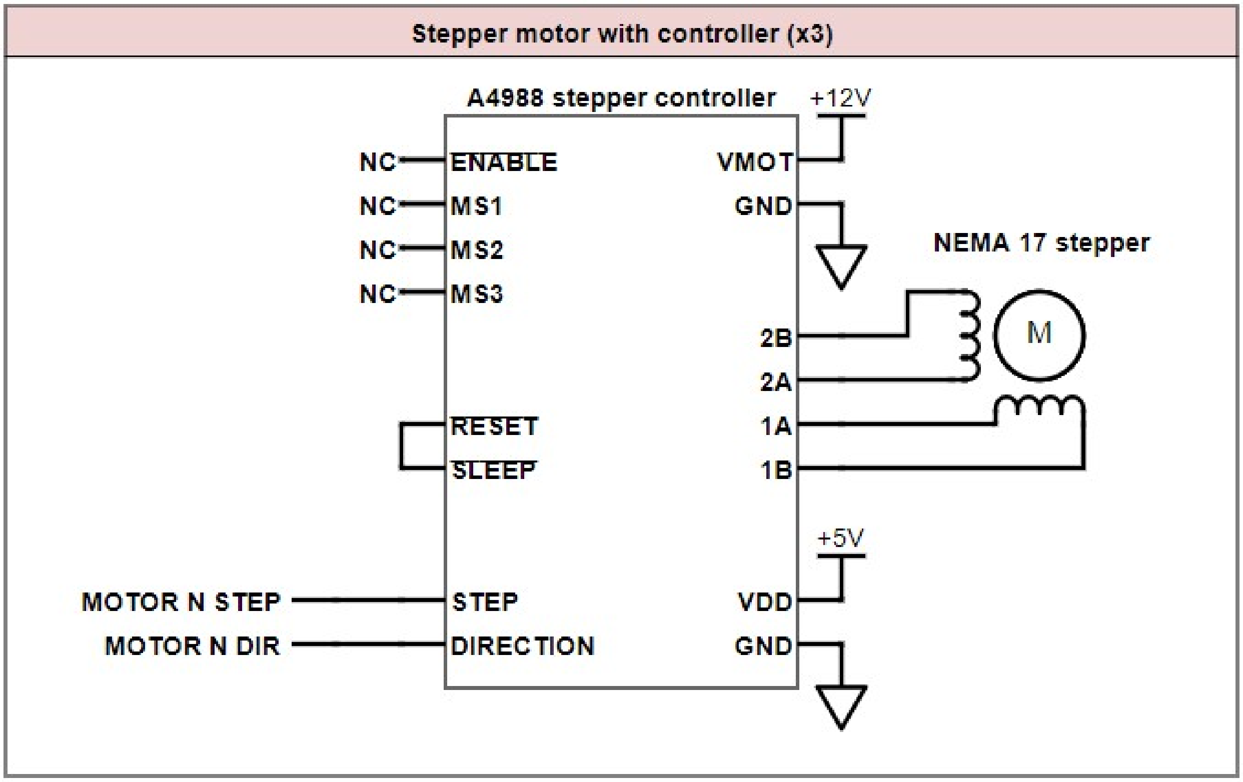

Figure C.5. Circuit diagram of a NEMA 17 stepper motor and its controller module. NC: no connection.

Supplementary Material D: CMAS Arduino Code

```
/*
  LAST UPDATE 2021-08-10
  VERSION 1.0

  Firmware for the Concentration Measurement and Adjustment System (CMAS.)
  Developed at the Aquatic Germplasm and Genetic Resources Center,
  Louisiana State University Agricultural Center, by Nikolas Zuchowicz.
  aquaticgermplasm.com
  nikolas.z@outlook.com

  Intended for use with an Arduino Nano in the assembled CMAS.

  ====

  LIBRARY ATTRIBUTIONS AND LICENSES

  AccelStepper.h is by Mike McCauley
(http://www.airspayce.com/mikem/arduino/AccelStepper/index.html)
  and is distributed under the GPL V3 license (GNU General Public License.)

  TimerOne.h is by Paul Stoffregen
(https://github.com/PaulStoffregen/TimerOne)
  and is distributed under the CC BY 3.0 US license (Creative Commons
Attribution 3.0.)
*/

#include <Wire.h>                     // General communication
#include <LiquidCrystal_I2C.h>        // LCD screen
#include <Adafruit_MCP4725.h>         // Digital-analog converter (DAC)
hardware
#include <SPI.h>                      // SPI communication protocol for SD
memory card
#include <SD.h>                       // SD memory card
#include <RTClib.h>                   // Real-time clock
#include <AccelStepper.h>             // Stepper motor control (for the
peristaltic pumps)
#include <TimerOne.h>                 // Stepper command scheduling

// Peristaltic pump motor control pins
#define PIN_MOTOR1_STEP 5
#define PIN_MOTOR1_DIR 0
#define PIN_MOTOR2_STEP 6
#define PIN_MOTOR2_DIR 7
#define PIN_MOTOR3_STEP 9
#define PIN_MOTOR3_DIR 8

// Analog-digital conversion input pins from the optics
#define PIN_OPTICALIN_1 A0
#define PIN_OPTICALIN_2 A1
#define PIN_OPTICALIN_3 A2
#define PIN_OPTICALIN_4 A3
#define PIN_OPTICALIN_5 A6

// Analog-digital conversion input pins from the optics
```

```
#define PIN_ROTENC_BUTTON 2
#define PIN_ROTENC_ROT1 3
#define PIN_ROTENC_ROT2 4

// Device select pin for the SD memory card
#define PIN_SD_SELECT 10

// I2C object instances
LiquidCrystal_I2C lcd(0x27, 20, 4);
Adafruit_MCP4725 opticaldac_1;
Adafruit_MCP4725 opticaldac_2;
Adafruit_MCP4725 opticaldac_3;
Adafruit_MCP4725 opticaldac_4;
Adafruit_MCP4725 opticaldac_5;
RTC_DS3231 rtc;

File SD_logfile;

// Voltage out for DACs to drive optics
int opticaldac_1_vout = 0;
int opticaldac_2_vout = 0;
int opticaldac_3_vout = 0;
int opticaldac_4_vout = 0;
int opticaldac_5_vout = 0;

// Voltage in from ADCs from optics
int opticaladc_1_vin = 0;
int opticaladc_2_vin = 0;
int opticaladc_3_vin = 0;
int opticaladc_4_vin = 0;
int opticaladc_5_vin = 0;

// State machine refresh timers
unsigned long currentMillis = 0;

unsigned long dac_previousMillis = 0;
const long dac_interval = 500;

unsigned long lcd_previousMillis = 0;
const long lcd_interval = 500;

unsigned long rtc_previousMillis = 0;
const long rtc_interval = 1000;

// Current cursor position on the LCD
int cursor_position = 0;

// Create stepper motor control objects for the three peristaltic pumps
AccelStepper motor1(AccelStepper::DRIVER, PIN_MOTOR1_STEP, PIN_MOTOR1_DIR);
AccelStepper motor2(AccelStepper::DRIVER, PIN_MOTOR2_STEP, PIN_MOTOR2_DIR);
AccelStepper motor3(AccelStepper::DRIVER, PIN_MOTOR3_STEP, PIN_MOTOR3_DIR);

// States for the finite state machine
enum state {ST_INIT, ST_MENU, ST_MANUAL_MOTORS, ST_SINGLE_CH_CONC,
SST_SINGLE_CH_CONC_ADJUST, ST_DILUTE_SAMP, ST_SYS_INFO};
enum state current_state = ST_INIT;
```

```
// Interrupt service routine flags
volatile bool flag_encoderButtonPressed = false;
volatile int flag_encoderRotated = 0;

void setup() {

  pinMode(PIN_ROTENC_BUTTON, INPUT);
  pinMode(PIN_ROTENC_ROT1, INPUT);
  pinMode(PIN_ROTENC_ROT2, INPUT);

  // Connect rotation and button push on the rotary encoder to interrupt
service routines
  attachInterrupt(digitalPinToInterrupt(PIN_ROTENC_BUTTON), encoderButtonISR,
RISING);
  attachInterrupt(digitalPinToInterrupt(PIN_ROTENC_ROT1), encoderRotISR,
RISING);

  // Initialize the five DACs with separate I2C addresses
  opticaldac_1.begin(0x60);
  opticaldac_2.begin(0x61);
  opticaldac_3.begin(0x62);
  opticaldac_4.begin(0x63);
  opticaldac_5.begin(0x64);

  opticaldac_1.setVoltage(0, false);
  opticaldac_2.setVoltage(0, false);
  opticaldac_3.setVoltage(0, false);
  opticaldac_4.setVoltage(0, false);
  opticaldac_5.setVoltage(0, false);

  // Initialize the LCD
  lcd.init();
  lcd.backlight();
  lcd.home();
  lcd.print("CMAS firmware v1.0");

  // Initialize the SD memory card
  lcd.setCursor(0, 1);
  if (!SD.begin(PIN_SD_SELECT)) {
    lcd.print("SD init failure");
    lcd.setCursor(0, 2);
    lcd.print("Check SD card");
  } else {
    lcd.print("SD init success");
  }

  lcd.setCursor(0, 3);
  if (!rtc.begin()) {
    lcd.print("Cannot find RTC");
  } else {
    DateTime time = rtc.now();
    lcd.print(time.timestamp());
  }

  delay(4000);
  lcd.clear();
```

```
}

void loop() {

  // The main loop runs a finite state machine.
  // The code for each state is devolved to a separate function.

  currentMillis = millis();

  switch (current_state) {

    case ST_INIT: // Initialization
      current_state = runstate_INIT();
      break;

    case ST_MENU: // Main menu to access other functions
      current_state = runstate_MENU();
      break;

    case ST_MANUAL_MOTORS: // Activate and deactivate pumps
      current_state = runstate_MANUAL_MOTORS();
      break;

    case ST_SINGLE_CH_CONC: // Measure input on a single optical channel
      current_state = runstate_SINGLE_CH_CONC();
      break;

    case SST_SINGLE_CH_CONC_ADJUST: // Adjust optical output on a single
optical channel
      current_state = runsubstate_SINGLE_CH_CONC_ADJUST();
      break;

    case ST_DILUTE_SAMP: // Run a dilution program (dilutes 1 sample fluid
with 1 extender fluid)
      current_state = runstate_DILUTE_SAMP();
      break;

    case ST_SYS_INFO: // Show system information, e.g. real time clock
timestamp
      current_state = runstate_SYS_INFO();
      break;

  }

}

state runstate_INIT() {

  // Maximum motor pulsetrain of 100 Hz, above which linear control breaks
down
  motor1.setMaxSpeed(100);
  motor1.setSpeed(0);
  motor2.setMaxSpeed(100);
  motor2.setSpeed(0);
  motor3.setMaxSpeed(100);
  motor3.setSpeed(0);
```

```
  // This timer interrupt runs once per millisecond to make sure that the
stepper motors run smoothly
  // and concurrently with other operations such as the I2C LCD refresh.
Without this, the motors
  // pause for the duration of each LCD refresh.

  // Set motor refresh to 1000 Hz
  Timer1.initialize(1000);
  Timer1.attachInterrupt(pollMotors);

  // DAC output voltage of (1580/4095)*5 = 1.93 V places the ADC input to
around 1.50 V.
  // This needs adjustment on the fly, but this is close to what is needed.
  opticaldac_1_vout = 1580;
  opticaldac_1.setVoltage(opticaldac_1_vout, false);

  transition_to_state_MENU();
  return ST_MENU;

}

void transition_to_state_MENU() {

  flag_encoderRotated = 0;

  lcd.clear();
  lcd.setCursor ( 0, 0 );
  lcd.print(" Run motors manually");
  lcd.setCursor ( 0, 1 );
  lcd.print(" Single-chan. conc.");
  lcd.setCursor ( 0, 2 );
  lcd.print(" Dilute sample");
  lcd.setCursor ( 0, 3 );
  lcd.print(" System info");

  lcd_refresh_cursor();

}

state runstate_MENU() {

  if (flag_encoderRotated != 0) {
    cursor_position += flag_encoderRotated;
    if (cursor_position < 0) cursor_position = 3;
    if (cursor_position > 3) cursor_position = 0;
    flag_encoderRotated = 0;
    lcd_refresh_cursor();
  }

  if (flag_encoderButtonPressed) {

    flag_encoderButtonPressed = false;
    switch (cursor_position) {
      case 0:
        transition_to_state_MANUAL_MOTORS();
        return ST_MANUAL_MOTORS;
      case 1:
```

```
        transition_to_state_SINGLE_CH_CONC();
        return ST_SINGLE_CH_CONC;
      case 2:
        transition_to_state_DILUTE_SAMP();
        return ST_DILUTE_SAMP;
      case 3:
        transition_to_state_SYS_INFO();
        return ST_SYS_INFO;
    }

  } else {
    return ST_MENU;
  }

}

void transition_to_state_MANUAL_MOTORS() {

  lcd.clear();
  lcd.setCursor ( 0, 0 );
  lcd.print(" Run motor 1");
  lcd.setCursor ( 0, 1 );
  lcd.print(" Run motor 2");
  lcd.setCursor ( 0, 2 );
  lcd.print(" Run motor 3");
  lcd.setCursor ( 0, 3 );
  lcd.print(" Exit to main menu");

  cursor_position = 0;
  lcd_refresh_cursor();

}

state runstate_MANUAL_MOTORS() {

  if (flag_encoderRotated != 0) {
    cursor_position += flag_encoderRotated;
    if (cursor_position < 0) cursor_position = 3;
    if (cursor_position > 3) cursor_position = 0;
    flag_encoderRotated = 0;
    lcd_refresh_cursor();
  }

  if (flag_encoderButtonPressed) {

    flag_encoderButtonPressed = false;
    switch (cursor_position) {
      case 0:
        if (motor1.speed() != 0) {
          motor1.setSpeed(0);
        } else {
          motor1.setSpeed(100);
        }
        break;
      case 1:
        if (motor2.speed() != 0) {
          motor2.setSpeed(0);
```

```
        } else {
          motor2.setSpeed(100);
        }
        break;
      case 2:
        if (motor3.speed() != 0) {
          motor3.setSpeed(0);
        } else {
          motor3.setSpeed(100);
        }
        break;
      case 3:
        cursor_position = 0; // sets correct cursor position in menu
        transition_to_state_MENU();
        return ST_MENU;
    }

  } else {
    return ST_MANUAL_MOTORS;
  }
}

void transition_to_state_SINGLE_CH_CONC() {

  lcd.clear();
  lcd.setCursor ( 0, 0 );
  lcd.print(" Single-chan. conc.");
  lcd.setCursor ( 0, 1 );
  lcd.print("  OUT           V");
  lcd.setCursor ( 6, 1 );
  lcd.print(opticaldac_1_vout);
  lcd.setCursor ( 11, 1 );
  lcd.print((float)opticaldac_1_vout / 4095 * 5);
  lcd.setCursor ( 0, 2 );
  lcd.print("  IN            V");

  cursor_position = 0;
  lcd_refresh_cursor();

}

state runstate_SINGLE_CH_CONC() {

  if (currentMillis - dac_previousMillis >= dac_interval) {
    dac_previousMillis += dac_interval;
    read_adcs();
    lcd.setCursor ( 6, 2 );
    lcd.print(opticaladc_1_vin);
    lcd.setCursor ( 11, 2 );
    lcd.print((float)opticaladc_1_vin / 1023 * 5);
  }

  if (flag_encoderRotated != 0) {
    cursor_position += flag_encoderRotated;
    flag_encoderRotated = 0;
    if (cursor_position < 0) cursor_position = 1;
    if (cursor_position > 1) cursor_position = 0;
```

```
    lcd_refresh_cursor();
  }

  if (flag_encoderButtonPressed) {
    flag_encoderButtonPressed = false;
    switch (cursor_position) {
      case 0:
        cursor_position = 1; // sets correct cursor position in menu
        transition_to_state_MENU();
        return ST_MENU;
        break;
      case 1:
        transition_to_substate_SINGLE_CH_CONC_ADJUST();
        return SST_SINGLE_CH_CONC_ADJUST;
        break;
    }
  } else {
    return ST_SINGLE_CH_CONC;
  }

}

void transition_to_substate_SINGLE_CH_CONC_ADJUST() {

  lcd.setCursor ( 1, 1 );
  lcd.print(">");

}

state runsubstate_SINGLE_CH_CONC_ADJUST() {

  if (currentMillis - dac_previousMillis >= dac_interval) {
    dac_previousMillis += dac_interval;
    read_adcs();
    lcd.setCursor ( 6, 2 );
    lcd.print(opticaladc_1_vin);
    lcd.setCursor ( 11, 2 );
    lcd.print((float)opticaladc_1_vin / 1023 * 5);
  }

  if (flag_encoderRotated != 0) {
    opticaldac_1_vout += flag_encoderRotated;
    flag_encoderRotated = 0;
    if (opticaldac_1_vout < 0) opticaldac_1_vout = 0;
    if (opticaldac_1_vout > 4095) opticaldac_1_vout = 4095;
    opticaldac_1.setVoltage(opticaldac_1_vout, false);
    lcd.setCursor ( 6, 1 );
    lcd.print(opticaldac_1_vout);
    lcd.setCursor ( 11, 1 );
    lcd.print((float)opticaldac_1_vout / 4095 * 5);
  }

  if (flag_encoderButtonPressed) {
    flag_encoderButtonPressed = false;
    transition_to_state_SINGLE_CH_CONC();
    cursor_position = 1;
    lcd_refresh_cursor();
```

```
    return ST_SINGLE_CH_CONC;
  } else {
    return SST_SINGLE_CH_CONC_ADJUST;
  }

}

void transition_to_state_DILUTE_SAMP() {

  lcd.clear();
  lcd.setCursor ( 0, 0 );
  lcd.print(" Dilute sample");

  cursor_position = 0;
  lcd_refresh_cursor();

}

state runstate_DILUTE_SAMP() {

  // Currently set with regression-based concentration calculation for an
algae sample
  long conc = (long)pow((float)10,((((float)opticaladc_1_vin / 1023 * 5)-
20.94)/(-2.9698)));
  lcd.setCursor ( 0, 1 );
  lcd.print(conc);
  lcd.print(" cells/mL");

  lcd.setCursor ( 0, 2 );
  if (conc <= 1000000) {
    lcd.print("Conc. too low to dilute");
  } else {
    int motor1_target;
    int motor2_target;

    if (conc <= 2000000) {
      motor1.setSpeed(100);
      motor2.setSpeed(((float)conc - (float)1000000) / (float)10000);

      lcd.print("P1 ");
      lcd.print(100);
      lcd.setCursor ( 0, 3 );
      lcd.print("P2 ");
      lcd.print((((float)conc - (float)1000000) / (float)10000));
    } else {
      motor1.setSpeed(((float)100 / (((float)conc - (float)1000000) /
(float)1000000)));
      motor2.setSpeed(100);

      lcd.print("P1 ");
      lcd.print(((float)100 / (((float)conc - (float)1000000) /
(float)1000000)));
      lcd.setCursor ( 0, 3 );
      lcd.print("P2 ");
      lcd.print(100);
    }
  }
```

```
  if (flag_encoderButtonPressed) {
    flag_encoderButtonPressed = false;

    motor1.setSpeed(0);
    motor2.setSpeed(0);

    cursor_position = 2; // sets correct cursor position in menu
    transition_to_state_MENU();
    return ST_MENU;
  } else {
    return ST_DILUTE_SAMP;
  }
}

void transition_to_state_SYS_INFO() {

  lcd.clear();
  lcd.setCursor ( 0, 0 );
  lcd.print(" Exit to main menu");
  lcd.setCursor ( 0, 1 );
  lcd.print(" Reset clock");
  lcd.setCursor ( 1, 2 );
  DateTime time = rtc.now();
  lcd.print(time.timestamp());

  cursor_position = 0;
  lcd_refresh_cursor();

}

state runstate_SYS_INFO() {

  if (currentMillis - rtc_previousMillis >= rtc_interval) {
    rtc_previousMillis += rtc_interval;
    lcd.setCursor ( 1, 2 );
    DateTime time = rtc.now();
    lcd.print(time.timestamp());
  }

  if (flag_encoderRotated != 0) {
    cursor_position += flag_encoderRotated;
    if (cursor_position < 0) cursor_position = 1;
    if (cursor_position > 1) cursor_position = 0;
    flag_encoderRotated = 0;
    lcd_refresh_cursor();
  }

  if (flag_encoderButtonPressed) {

    flag_encoderButtonPressed = false;
    switch (cursor_position) {
      case 0:
        cursor_position = 3; // sets correct cursor position in menu
        transition_to_state_MENU();
        return ST_MENU;
        break;
```

```
      case 1:
        rtc.adjust(DateTime(F(__DATE__), F(__TIME__)));
        break;
    }

  } else {
    return ST_SYS_INFO;
  }

}

void encoderButtonISR() {
  flag_encoderButtonPressed = true;
}

void encoderRotISR() {
  if (digitalRead(PIN_ROTENC_ROT1) == digitalRead(PIN_ROTENC_ROT2)) {
    flag_encoderRotated++;
  } else {
    flag_encoderRotated--;
  }
}

void lcd_refresh_cursor() {

  lcd.setCursor ( 0, 0 );
  (cursor_position == 0) ? lcd.print(">") : lcd.print(" ");
  lcd.setCursor ( 0, 1 );
  (cursor_position == 1) ? lcd.print(">") : lcd.print(" ");
  lcd.setCursor ( 0, 2 );
  (cursor_position == 2) ? lcd.print(">") : lcd.print(" ");
  lcd.setCursor ( 0, 3 );
  (cursor_position == 3) ? lcd.print(">") : lcd.print(" ");

}

void pollMotors() {

  motor1.runSpeed();
  motor2.runSpeed();
  motor3.runSpeed();

}

void read_adcs() {
  opticaladc_1_vin = analogRead(PIN_OPTICALIN_1);
  opticaladc_2_vin = analogRead(PIN_OPTICALIN_2);
  opticaladc_3_vin = analogRead(PIN_OPTICALIN_3);
  opticaladc_4_vin = analogRead(PIN_OPTICALIN_4);
  opticaladc_5_vin = analogRead(PIN_OPTICALIN_5);
}

void log_to_SD() {

  SD_logfile = SD.open("test1.txt", FILE_WRITE);

  if (SD_logfile) {
```

```
    SD_logfile.print(currentTime());
    SD_logfile.println("");
    SD_logfile.close();
  } else {
    lcd.clear();
    lcd.print("SD file error");
    delay(1000);
    lcd.clear();
  }
}

String currentTime() {
  String currentTime;
  DateTime now = rtc.now();
  currentTime = now.unixtime();
  return currentTime;
}
```

Supplementary Material E: Figures

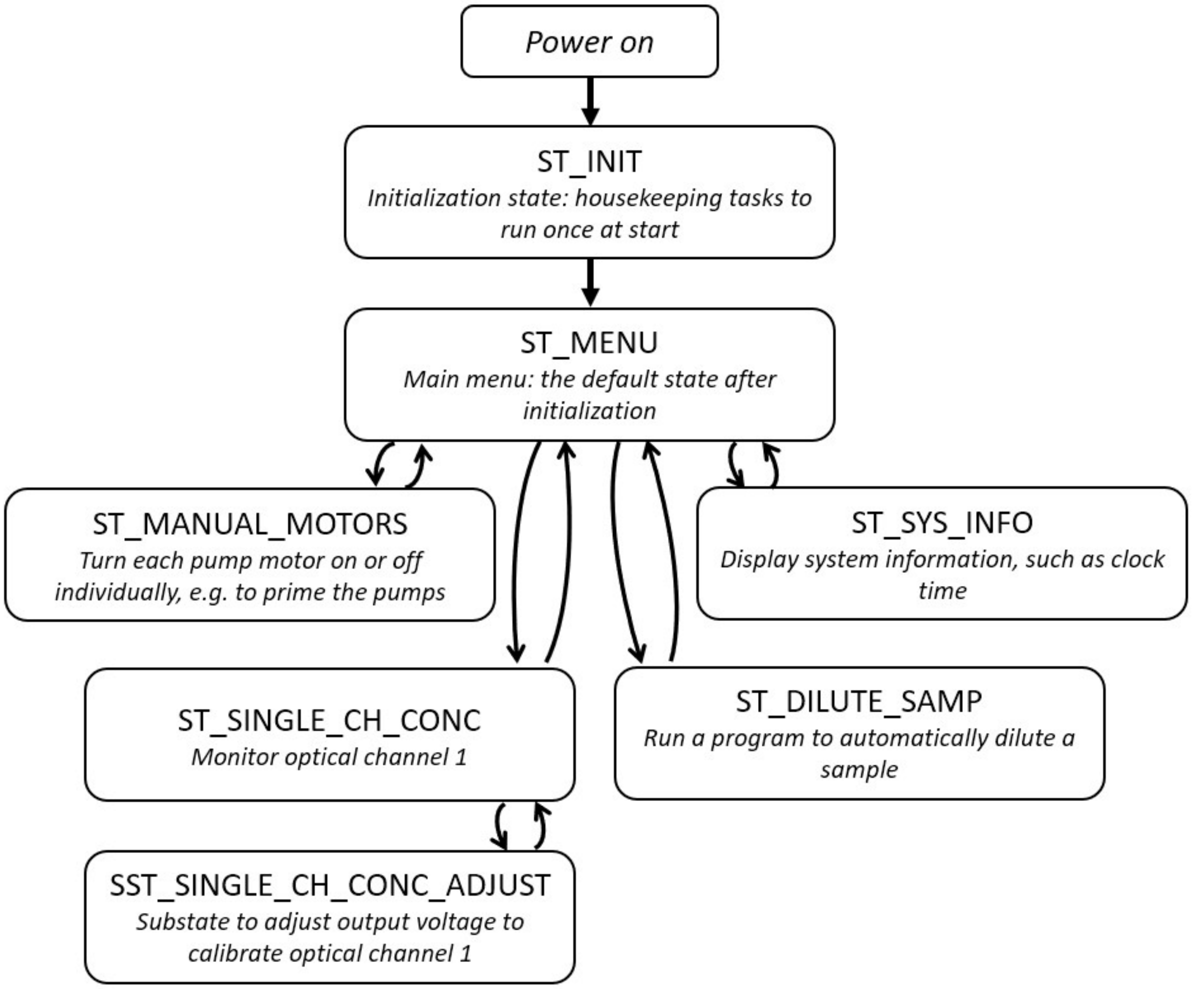

Figure E.1. The finite-state machine logic flow that controlled the CMAS. Capital letters indicate the names of states (ST) and substates (SST) as used in the Arduino firmware. Arrows indicate permitted transitions between states. For example, the firmware may transition directly from ST_SYS_INFO to ST_MENU, but not from ST_SYS_INFO to ST_DILUTE_SAMP, except via ST_MENU.

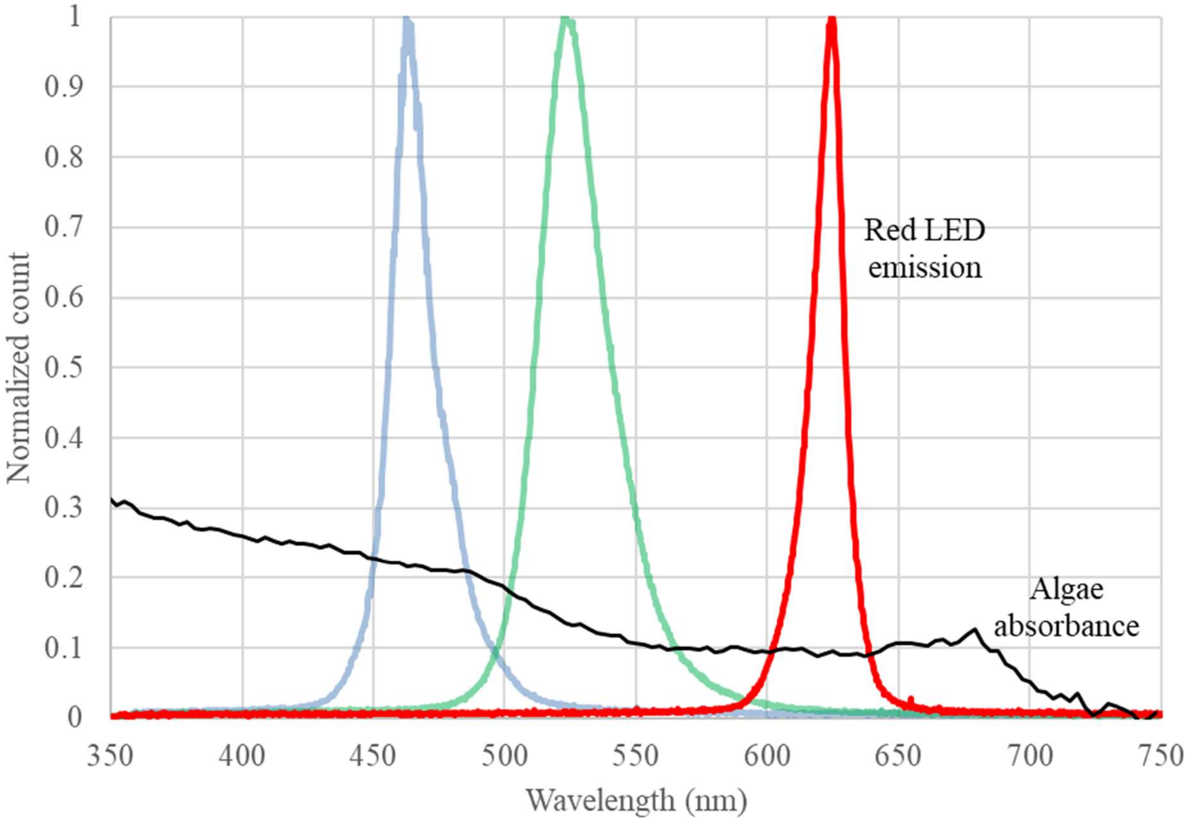

Figure E.2. Spectral absorbance of a sample of the algae *Tetraselmis chuii* (3.6×10^6 cells/mL) measured on a Nanodrop 1000 spectrophotometer. The measured emission distributions of the tri-color LED used in the CMAS are overlaid for comparison, with each distribution normalized to a maximum value of 1. All distributions are normalized (peak value = 1, $\lambda_{max,blue} = 462$ nm, $\lambda_{max,green} = 523$ nm, $\lambda_{max,red} = 624$ nm). The peak absorbance wavelength of the algal sample is not shown ($\lambda = 229$ nm).

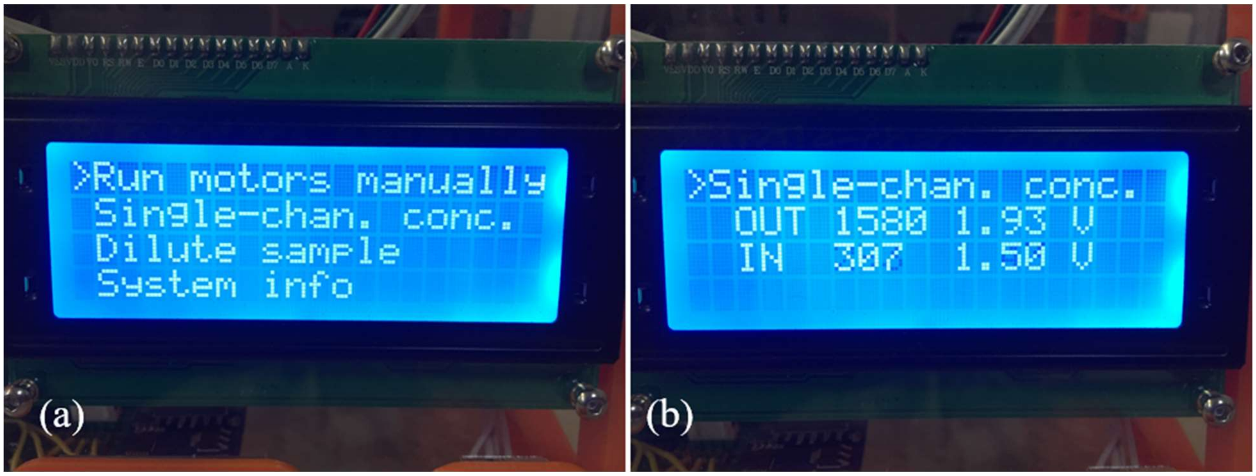

Figure E.3. An LCD screen provided feedback and estimation data to the user. (a) The main menu displayed in state ST_MENU (as shown in Fig. E.1). (b) Display of the output (DAC, LED control) and input (ADC, sample measurement) voltage signals associated by a single optical module.

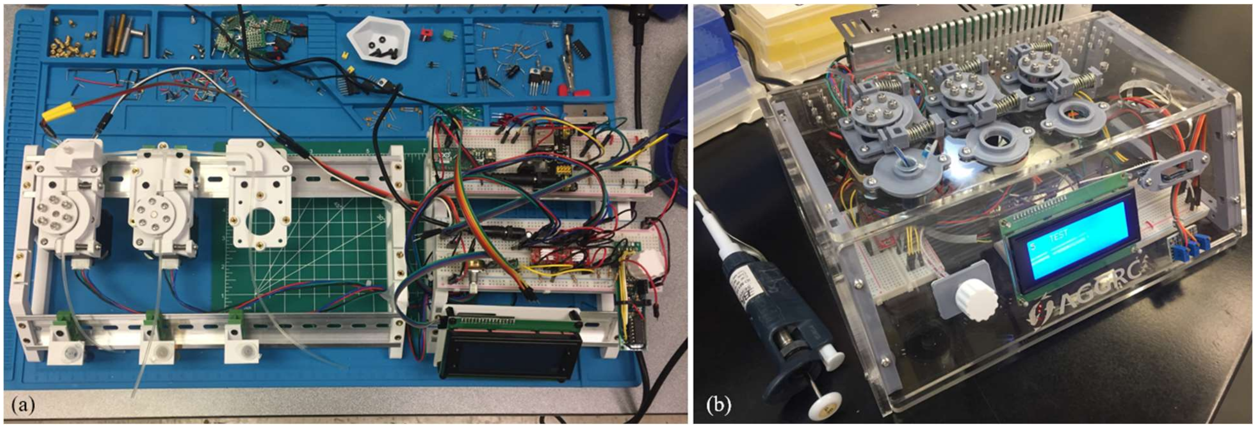

Figure E.4. (a) First prototype of the CMAS. Pumps and other electromechanical components were mounted on a frame made with aluminum DIN mounting rails. (b) Second prototype. A robust frame was fabricated from acrylic sheet and 3-D printed structural elements.

Supplementary Material F: 3-D Printer Settings

All parts were printed on an Ultimaker 3 Extended 3-D printer (Ultimaker, Utrecht, Netherlands). Slicing was performed in Ultimaker Cura software version 4.9.0. Settings were as given in the tables below.

Table F.1. Custom 3-D printer settings. All printer settings were the default for the "Normal – 0.15 mm" configuration except those given here.

**Setting**	**Value**
Initial layer height	0.15 mm
Support placement	Touching build plate
Support overhang angle	50°
Support density	20%
Support Z distance	0.0 mm
Support X/Y distance	0.2 mm
Minimum support X/Y distance	0.0 mm
Minimum support interface area	3.0 mm^2
Build plate adhesion type	Raft

Table F.2. Nozzle-specific settings.

	**Nozzle 1**	**Nozzle 2**
Nozzle type	AA 0.4 mm	AA 0.4 mm
Filament	Generic PLA	Generic Breakaway
Role	Build material	Support material
Initial layer line width	100%	120%
